# Injectisome assembly primes *Pseudomonas aeruginosa* for type III secretion

**DOI:** 10.1128/mbio.00545-26

**Published:** 2026-04-30

**Authors:** Kristen Ramsey, Shoichi Tachiyama, Apolline Brossard, Zhao Hang, Jun Liu, Barbara I. Kazmierczak

**Affiliations:** 1 Program in Microbiology, Yale University 5755, New Haven, Connecticut, USA; 2 Department of Microbial Pathogenesis, Yale University 5755, New Haven, Connecticut, USA; 3 Microbial Sciences Institute, Yale University 5755, New Haven, Connecticut, USA; 4 Department of Molecular, Cellular and Developmental Biology, Yale College 265053, New Haven, Connecticut, USA; 5 Department of Medicine, Yale University 5755, New Haven, Connecticut, USA; NYU Langone Health, New York, New York, USA

**Keywords:** phenotypic heterogeneity, type 3 secretion system, *Pseudomonas aeruginosa*, cAMP, type IV pili, virulence gene regulation, bistability

## Abstract

Many Gram-negative pathogens, including *Pseudomonas aeruginosa*, use a type III secretion system (T3SS) to intoxicate eukaryotic cells. The T3SS is an important virulence factor linked to increased morbidity and mortality in infections, yet its expression slows bacterial growth and activates innate immune receptors. T3SS genes are expressed heterogeneously, with T3SS-ON cells arising from “primed” bacteria that express the T3SS transcriptional activator ExsA and respond immediately to T3SS activating signals. However, the mechanistic basis for priming is not known. ExsA is part of a complex protein-sequestration network, positively regulating its own expression, as well as that of its anti-activator (ExsD), its anti-anti-activator (ExsC), and ExsC’s binding partner (ExsE). These four proteins create a bistable regulatory network. We hypothesized that transcription from a cAMP-dependent promoter upstream of ExsA could drive cells into the primed state, and tested this at the single-cell level. Exogenous cAMP increased the proportion of primed, ExsA-expressing cells, with whole-cell cryo-electron tomography demonstrating the assembly of T3SS injectisomes under these conditions. Interstrain variation in endogenous cAMP levels correlated with strain-specific proportions of primed bacteria, while genetic manipulation of cAMP levels altered primed population size. This work demonstrates how endogenous and exogenous cAMP inputs into a bistable regulatory switch generate subpopulations of T3SS-primed cells poised to respond to activating signals.

**IMPORTANCE:**

Type III secretion systems (T3SS) are specialized protein secretion systems that allow bacteria to inject toxins into eukaryotic cells. T3SS are important virulence factors, but their expression carries a fitness cost: they slow bacterial growth and make bacteria vulnerable to detection by the innate immune system. Some pathogens, like *Pseudomonas aeruginosa,* balance the costs and benefits of T3SS expression by restricting T3SS expression to a subset of cells. T3SS-ON cells arise from “primed” bacteria that express the transcriptional activator ExsA and respond immediately to T3SS activating signals. However, the mechanistic basis for priming is unknown. In this study, we tested whether expression of ExsA from a cAMP-dependent promoter could drive cells into the primed state and found this to be true. Whole-cell cryo-electron tomography demonstrated that primed bacteria assembled T3SS injectisomes. This work demonstrates how cAMP inputs into a bistable regulatory switch generate subpopulations of T3SS-primed cells.

## INTRODUCTION

Genetically identical cells, be they bacteria or cancer cells, exhibit non-identical behaviors (1, 2). Such phenotypic heterogeneity can arise from stochastic variation in gene expression that is then propagated and amplified by regulatory networks, yielding distinct subpopulations of cells (2–4). The generation of phenotypically diverse cells (e.g., antibiotic persisters) can increase adaptive fitness in unpredictable, fluctuating environments (bet-hedging) or enable a division of labor between subpopulations producing beneficial yet costly shared goods (5, 6). Among pathogenic bacteria, heterogeneous expression of the type III secretion system (T3SS) leads to “cooperative virulence,” allowing populations to balance the individual fitness cost of T3SS expression with the shared benefits of niche creation, barrier disruption, and immune defense (7–9).

The T3SS injectisome is a virulence-associated nanomachine that directly injects bacterial effector proteins into host cells. This needle-like structure is composed of ~20 proteins that are conserved among many Gram-negative pathogens and symbionts. Cryo-electron microscopy (cryo-EM) and cryo-electron tomography (cryo-ET) studies have revealed that the injectisome is composed of a cytoplasmic complex, sorting platform, envelope-spanning basal body, needle, and needle tip complex (10–14). When the needle tip complex contacts a host cell membrane, the translocon complex is recruited at the tip, forming a channel into the host cell (15). Translocated effectors then target a variety of host cell functions, which promote bacterial survival, inhibit immune activation, and often result in host cell death. In the opportunistic pathogen *Pseudomonas aeruginosa*, the injectisome targets eukaryotic cells ranging from predatory amoeba to human phagocytes and epithelial cells, leading to cytoskeletal disruption and cell death (16–20). The T3SS injectisome is critical for *P. aeruginosa* to establish acute murine and human infection and is linked with increased morbidity and mortality in hospital-acquired infections (21, 22).

Single-cell studies have established that *P. aeruginosa* heterogeneously expresses T3SS genes, yielding bimodal populations of “OFF” and “ON” cells in the presence of activating signals (23–27). T3SS-ON cells grow slowly, while recognition of T3SS components by NLRC4 inflammasomes may incur additional fitness costs during infection (26, 28, 29). Nonetheless, ExoU, a phospholipase A2 injected by the T3SS, rapidly kills recruited neutrophils and macrophages and serves as a shared public good in murine infections (20, 23). Thus, *P. aeruginosa* may use phenotypic heterogeneity to maintain the T3SS+ genotype while avoiding the measurable fitness costs associated with expressing this virulence trait (23, 26).

The AraC-family transcriptional activator, ExsA, controls the entire *P. aeruginosa* T3SS regulon, which encodes the needle complex, secreted effectors, and ExsA regulators (30, 31). Upregulation of ExsA-dependent gene expression is coupled to activation of the T3SS injectisome via a “partner-switching” model that assumes the presence of an assembled T3SS needle. In the absence of activating signals, the needle is closed and incapable of secretion; intracellular ExsA is bound by its anti-activator ExsD, and ExsC is sequestered by ExsE (32, 33). Activation signals, such as host cell contact or *in vitro* Ca^2+^ chelation, induce translocon formation and allow ExsE secretion, freeing ExsC to bind ExsD (33–35). This allows ExsA to dimerize and promote transcription of the T3SS regulon (32, 36). Such a model raises the question, however, of how T3SS genes are expressed to allow assembly of the injectisome in the first place.

Using microscopy and single-cell lineage tracking, we previously established that, naïve of any exogenous T3SS-activating signal, some *P. aeruginosa* cells express ExsA (26). When exposed to a T3SS-activating signal, these ExsA-positive cells rapidly give rise to T3SS-expressing cells in the population, behaving as if primed to respond. In this study, we examined how priming arises and whether it is correlated with T3SS injectisome assembly. We also investigated the role of the second messenger cyclic-AMP (cAMP), which activates a Vfr-cAMP-dependent promoter upstream of *exsA* (P*_exsA_*) that drives ExsA expression but not that of ExsD, ExsC, or ExsE (25). cAMP is a well-established positive regulator of *P. aeruginosa* T3SS gene expression: deletion of genes encoding *vfr*, the adenylate cyclases *cyaA* and/or *cyaB*, or P*_exsA_* itself significantly impairs T3SS gene expression and reduces *P. aeruginosa* virulence (25, 37, 38). Given the importance of cAMP in T3SS-dependent virulence, we chose to test its role in generating heterogeneity in T3SS priming and expression.

## RESULTS

### *P. aeruginosa* assembles an injectisome in the absence of T3SS-activating signals

**Fig 1:**
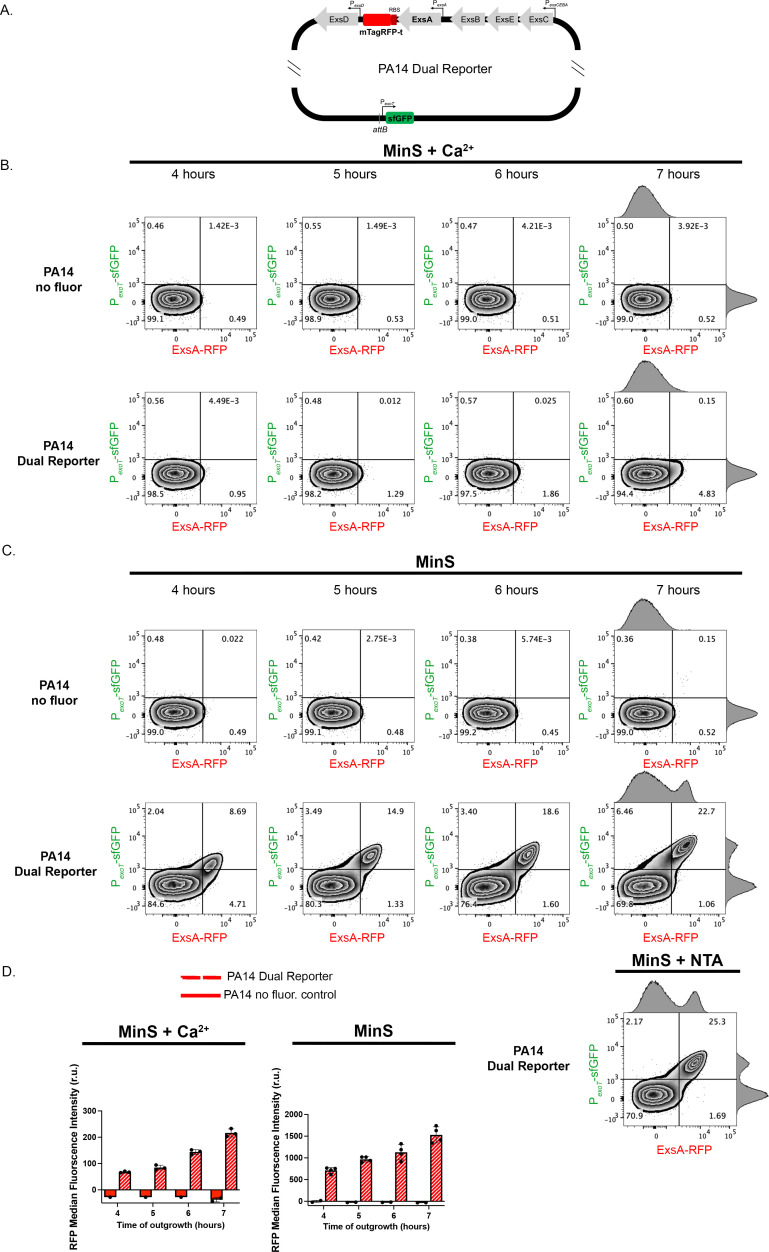
An ExsA+, T3SS-primed subpopulation of cells is identifiable by flow cytometry. (**A**) Schematic of dual *exsA*-IRES-*mTagRFP-t attB*::P*_exoT_-sfGFP* reporter construct. Representative flow cytometry zebra plots (showing combined contour and density) measuring ExsA production (red, x-axis) and ExoT transcription (green, y-axis) in PA14 WT no-fluor control (top) or PA14 Dual ExsA/T3SS reporter cells (bottom) grown planktonically in (**B**) T3SS secretion-repressing MinS + Ca^2+^ media or (**C**) T3SS secretion-activating MinS or MinS NTA media and sampled at 4–7 h. Adjunct histograms for RFP and GFP fluorescence intensities are shown for the 7-h timepoint. Quadrants were established using a WT PA14 no-fluor control and validated on a 1:1 mixture of single GFP+ and RFP+ PA14 samples. (**D**) Quantification of RFP median fluorescence intensities over time for either the PA14 dual reporter (red stripe) or the PA14 no fluorescent reporter control (solid red) in MinS + Ca^2+^ (left) and MinS (right).

Lineage tracking studies of *P. aeruginosa* PA14 cells carrying chromosomal reporters for both ExsA production (*exsA*-IRES-*mTagRFP-t*) and ExsA-dependent T3SS gene transcription (*attB*::P*_exoT_*-sfGFP) (Fig. 1A) established that a primed ExsA+/RFP+ subpopulation of cells is present even in the absence of T3SS-activating signals (e.g., Ca^2+^ replete conditions), and that these cells quickly respond to activating signals (such as the chelator nitriloacetic acid [NTA]) by inducing T3SS gene expression (26). We confirmed our ability to detect these populations by flow cytometry (Fig. S3), again observing a subpopulation of ExsA+/RFP+ cells which remained T3SS−/GFP− when cultured planktonically in MinS media supplemented with Ca^2+^ (Fig. 1B). We estimated this population’s size by gating on a reporterless PA14 “no fluor” control and observed fewer ExsA+ RFP+ cells (5%) than we had observed by microscopy (10%–14%) (26). This was likely due to the dimness of this chromosomal reporter’s signal, which we imaged with extended exposures during microscopy. When the dual reporter strain was grown in T3SS-activating media (either MinS or MinS + NTA), a bimodal distribution of RFP−/GFP− and RFP+/GFP+ cells was observed, corresponding to the T3SS-OFF and T3SS-ON cells previously seen by microscopy and lineage-tracking (Fig. 1C) (26). RFP fluorescence (MFI) was much brighter in bacteria cultured in MinS compared to MinS + Ca^2+^, which was consistent with ongoing *exsCEBA* expression in these T3SS-activated conditions (Fig. 1D); this also increased the proportion of cells gated as ExsA+/RFP+ (20%–25%). Western blotting confirmed that effector and translocon proteins were secreted by bacteria cultured in MinS or MinS + NTA, but not MinS + Ca^2+^, while cell-associated PopB and PopD were detected in all conditions, including MinS + Ca^2+^ (Fig. S4) (39).

**Fig 2:**
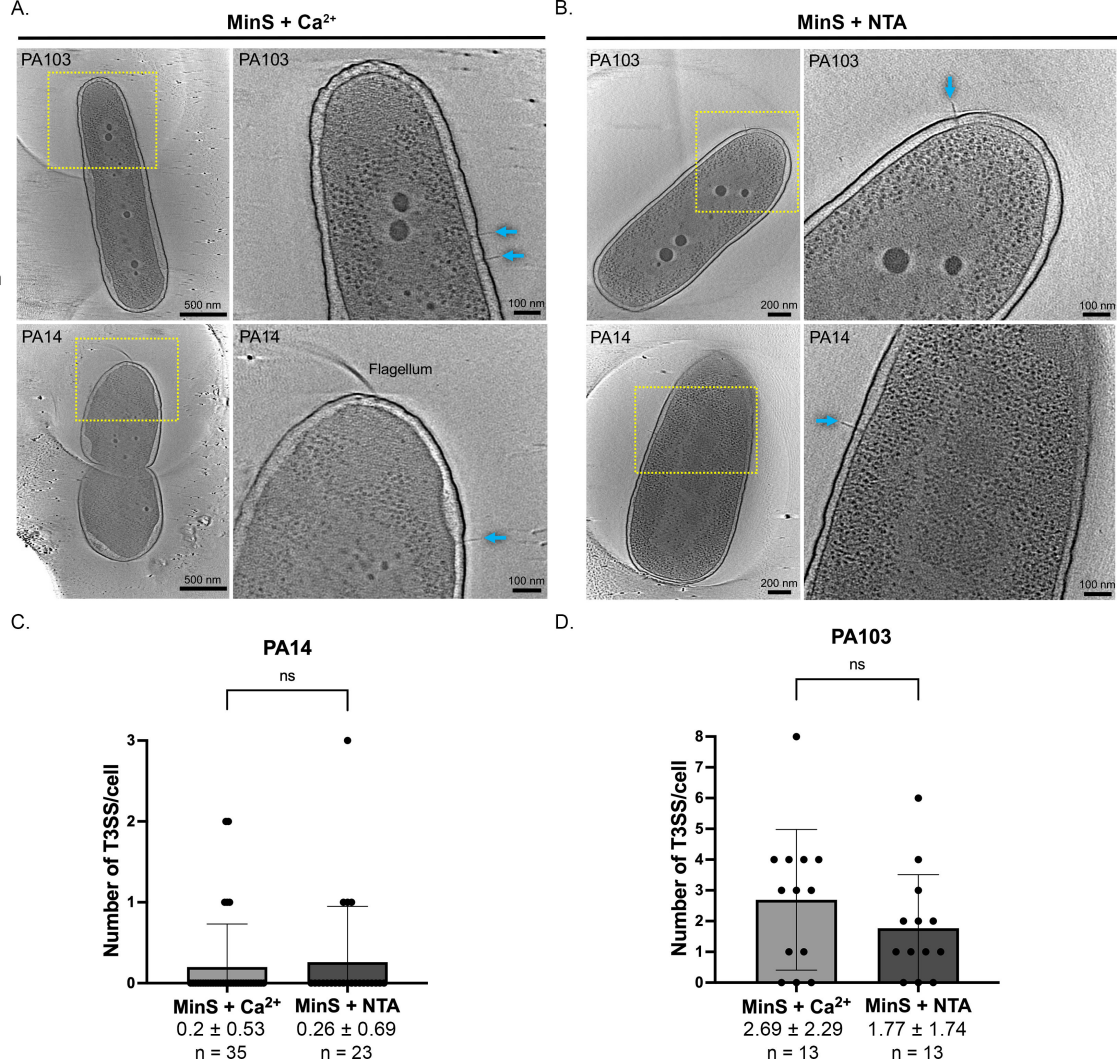
T3SS injectisome assembly is not dependent on an activation signal. PA103 and PA14 WT cells were grown in (**A**) MinS + Ca^2+^ or (**B**) MinS + NTA for 7 h, then prepared and imaged by cryo-ET at a magnification of 6.184 Å physical pixel size. Injectisomes on the bacterial surface were counted manually and are indicated by blue arrows. Representative tomograms are shown. (**C**) Quantification of PA14 T3SS needles per cell in both growth conditions. (**D**) Quantification of PA103 T3SS needles per cell in both growth conditions. Bars show mean ± SD. Significance determined by Welch’s unequal variances t-test; ns, not significant.

ExsA overexpression is sufficient to induce the expression of genes encoding the T3SS apparatus, effectors, and regulators (37). We wondered whether injectisome assembly occurred under T3SS-repressing conditions, where we observed ExsA+/RFP+ cells. To test this, we visualized surface structures of live *P. aeruginosa* with whole-cell cryo-ET, employing strains PA14 and PA103; this latter strain has a higher proportion of T3SS-ON cells than PA14 (26). We could readily identify and count needles (Fig. 2A and B; Fig. S5); subtomogram averaging of these needle-like complexes allowed us to determine *in situ* structures and confirm visualization of injectisomes with basal bodies in the periplasm (Fig. S5). When grown in MinS + Ca^2+^, 5 of 35 (14%) PA14 cells and 10 of 13 (77%) PA103 cells had one or more T3SS needles as observed by whole-cell cryo-ET (Fig. 2C and D). PA103 also had more needles per cell (mean, 2.7 ± 2.3) than PA14. The proportion of cells with assembled injectisomes remained about the same when either PA14 (17%) or PA103 (77%) was cultured in T3SS-activating MinS + NTA media (Fig. 2C and D). Although we could not resolve cytoplasmic components of the injectisome by the subtomogram averaging, interrogation of PA103 carrying transcriptional reporters for each T3SS regulatory and structural gene operon (P*_exsC_-lux*, P*_exsD_-lux*, P*_pscN_-lux*, P*_pcrG_-lux*, P*_popN_-lux*) in MinS + Ca^2+^ showed that each operon was expressed under non-activating conditions where T3SS injectisomes were seen (Fig. S6). Expression was absolutely dependent on ExsA. In contrast, a reporter for the T3SS effector ExoU showed minimal expression in MinS + Ca^2+^ under these non-activating conditions, consistent with the low expression of P*_exoT_*-sfGFP in dual reporter cells as observed by flow cytometry (Fig. 2B).

### cAMP increases the proportion of both ExsA+ cells and injectisome-positive cells

**Fig 3:**
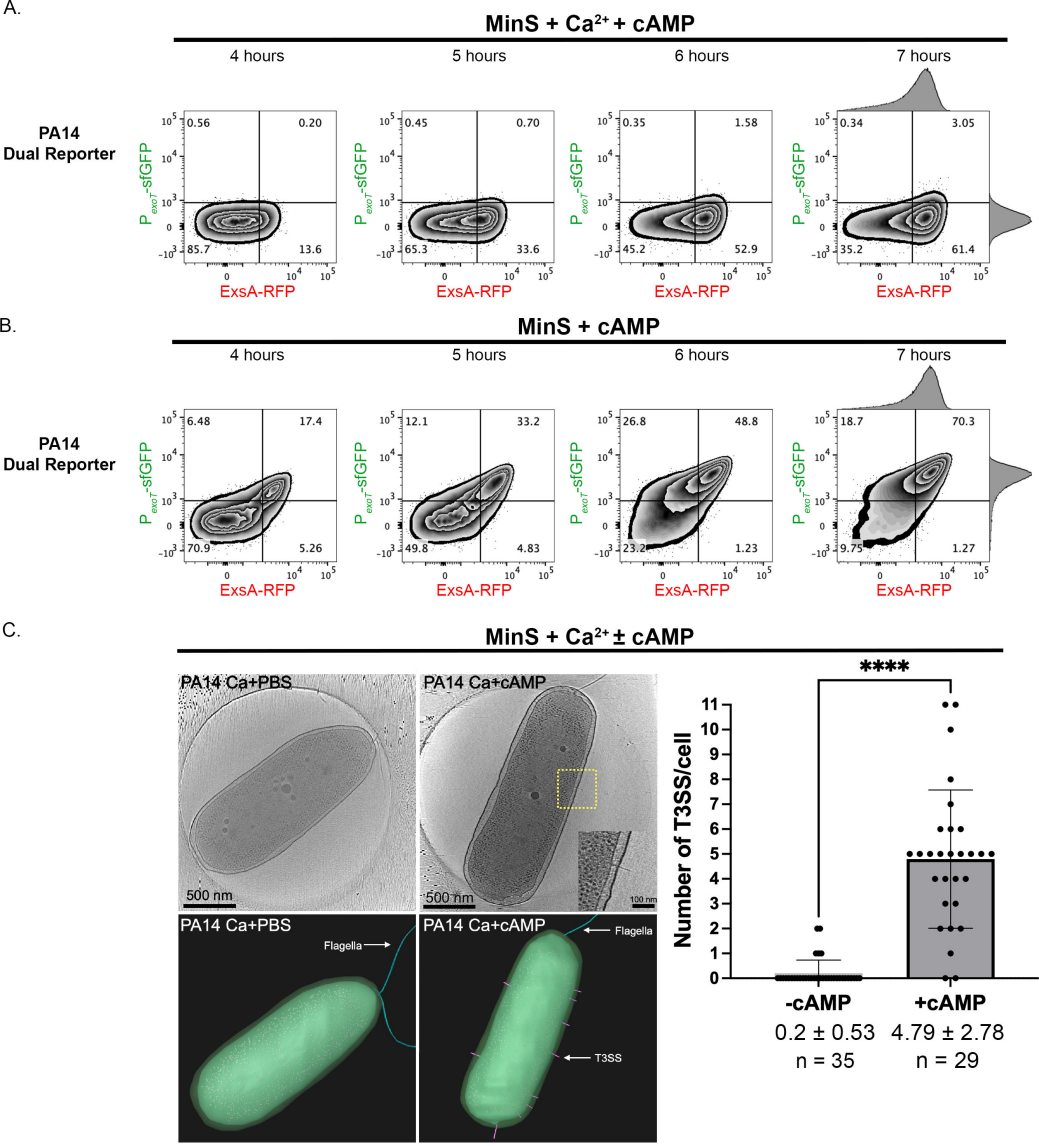
Exogenous cAMP increases the proportion of primed cells and assembled injectisomes**.** Representative flow cytometry zebra plots measuring ExsA production (red, x-axis) and ExoT transcription (green, y-axis) for PA14 dual reporter cells grown planktonically in (**A**) MinS + Ca^2+^ + 20 mM cAMP or (**B**) MinS + 20 mM cAMP media and sampled at 4-7 h. Adjunct histograms for RFP and GFP fluorescence intensities are shown for the 7-h timepoint. (**C**) Example tomogram of a PA14 cell grown in MinS + Ca^2+^ (left) or MinS + Ca^2+^ + 20 mM cAMP (right) for 7 h, then prepared and imaged by cryo-ET at a magnification of 6.184 Å physical pixel size. Inset: magnification of a representative injectisome in additional structural detail. 3D composite tomograms of each cell are shown below, with the T3SS injectisome pseudocolored pink. Quantification of T3SS injectisomes per cell grown in MinS + Ca^2+^ in the absence (left) or presence (right) of cAMP. (MinS + Ca^2+^ quantification reproduced from Fig. 2C.) Significance determined by Welch’s unequal variances t-test. Bars show mean ± SD. **** = *P*-value < 0.0001.

**Fig 4:**
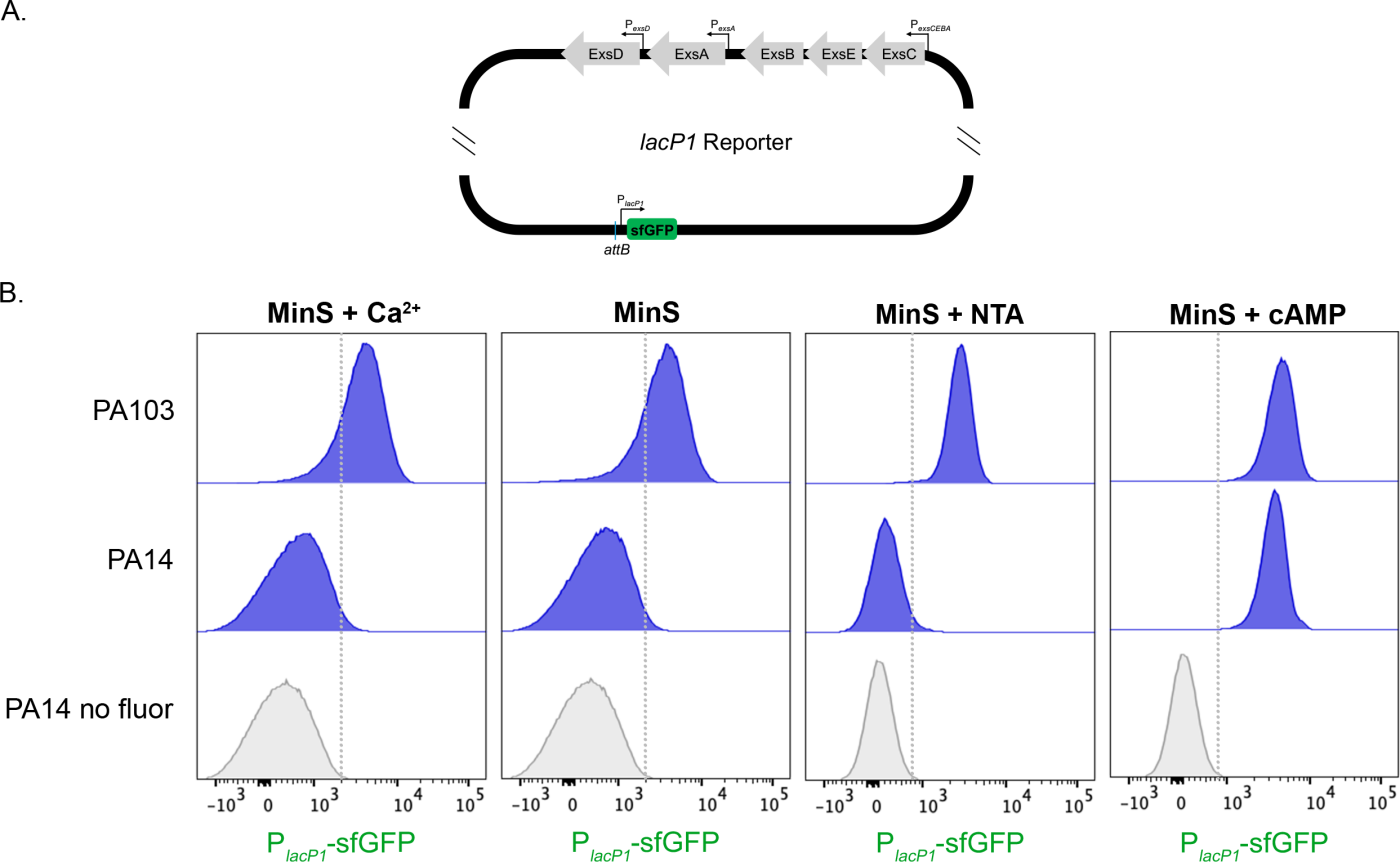
Strain variation in endogenous cAMP levels correlates with injectisome-positive cells**.** (**A**) Diagram of *P. aeruginosa* carrying the cAMP-responsive *attB*::P*_lacP1_*-sfGFP reporter. (**B**) PA14 and PA103 cells expressing P*_lacP1_*-sfGFP were cultured planktonically for 7 h in MinS + Ca^2+^, MinS, MinS + NTA, or a control of MinS + cAMP, fixed, and analyzed for GFP fluorescence by flow cytometry, with a fluorescent gate established using a *P. aeruginosa* PA14 control with no fluorescent reporter (gray histogram/line).

**Fig 5:**
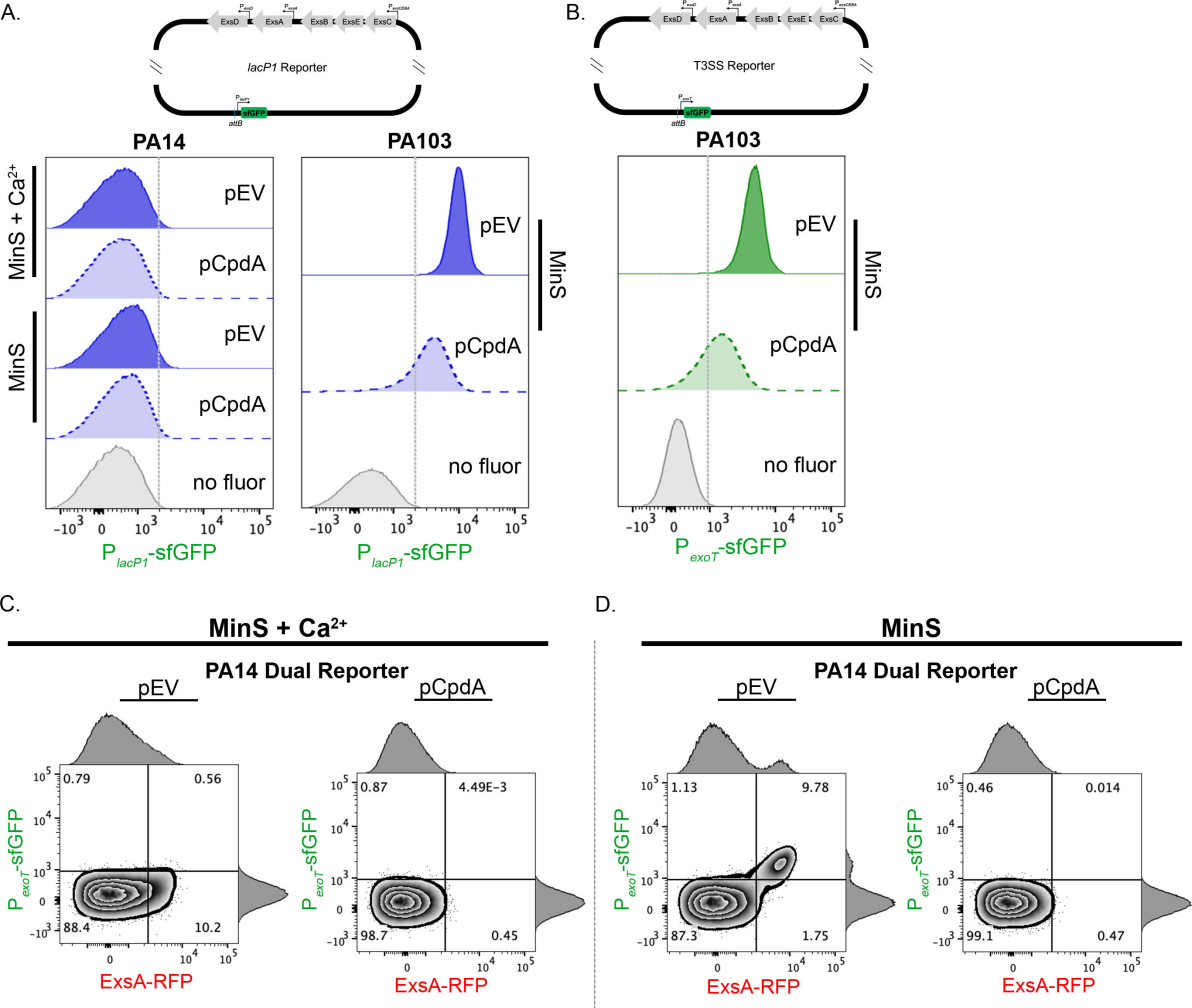
Breakdown of cAMP decreases T3SS-priming. (**A**) pMQ72 (“EV”) or pMQ72-CpdA (“pCpdA”) plasmids were introduced into PA14 and PA103 *attB*::P*_lacP1_*-sfGFP cAMP reporter strains; these were grown planktonically for 7 h in the indicated MinS + Ca^2+^ or MinS media, then analyzed for GFP fluorescence by flow cytometry. (**B**) PA103 carrying the *attB*::P*_exoT_*-sfGFP T3SS reporter with either pCpdA or pEV was grown in MinS and analyzed for GFP fluorescence as in panel **A**. GFP+ gating was established for both panels **A** and **B** based on isogenic no-fluor controls (gray line). PA14 *exsA*-IRES-*mTagRFP-t attB*::P_exoT_-*sfGFP* dual reporter bacteria carrying pEV or pCpdA were cultured planktonically in (**C**) MinS + Ca^2+^ or (**D**) MinS for 7 h and analyzed for RFP (*x*-axis) and GFP (*y*-axis) fluorescence, with adjunct fluorescence histograms shown on the zebra plot. Quadrants were established using a WT PA14 no-fluor control.

Prior work has shown that increasing cAMP, either by genetic manipulation or exogenous addition, increases *P. aeruginosa* T3SS expression (37, 40, 41). We tested whether cAMP also affected priming, using the dual reporter strain and flow cytometry. In MinS + Ca^2+^, cAMP treatment increased the proportion of ExsA+/RFP+ cells, consistent with its known activity at the P*_exsA_* promoter (Fig. 3A). Expression of the P*_exoT_* transcriptional reporter remained low in the absence of T3SS-activating signals. In T3SS-activating MinS, cAMP also increased the percentage of ExsA+/RFP+ cells, with P*_exoT_* expression increasing in tandem (Fig. 3B). Thus, cAMP treatment was sufficient to increase the proportion of ExsA-expressing cells, but an activating signal was still required for upregulation of T3SS expression. Contour plots of cells growing in MinS + cAMP showed a shift from a bimodal population (Fig. 3B, 4 and 5h) to a unimodal population that was RFP+/GFP+ by 6–7 h; however, some cells still fell into a quadrant that was gated as RFP−/GFP+ at these time points. As mTagRFP-t maturation takes ~87 min, while sfGFP matures more quickly (15.3 min)—and lineage tracking established a mean lag time of 89 min between ExsA and P*_exoT_* expression in such T3SS activating conditions—these results are consistent with cells exhibiting dim RFP signals despite producing ExsA for some time (26).

We next examined whether the increase in ExsA+/RFP+ cells following cAMP treatment was associated with more cells expressing injectisomes under non-activating conditions. Whole-cell cryo-ET demonstrated that most (93%) PA14 cells cultured in MinS + Ca^2+^ plus cAMP assembled injectisomes, with an average of 4.8 needles per cell (Fig. 3C). Thus, cAMP is sufficient to drive PA14 cells cultured in the absence of T3SS-activating signals to both express ExsA and assemble injectisomes. Interrogation of T3SS operon transcriptional reporters under these same conditions (MinS + Ca^2+^ plus cAMP) demonstrated increased expression of regulatory and structural operons in response to cAMP; again, transcription was absolutely dependent on ExsA (Fig. S6).

### Mutations that alter endogenous cAMP levels change the proportion of primed and T3SS-ON cells

The proportion of PA14 versus PA103 bacteria assembling injectisomes in the absence of cAMP treatment differed greatly; we wondered whether this reflected interstrain differences in endogenous cAMP levels. We assessed this by integrating a chromosomal cAMP-responsive *attB*::P*_lacP1_*-sfGFP transcriptional reporter in PA103 and PA14 (41) and measured fluorescence by flow cytometry. The P*_lacP1_* reporter responded robustly to exogenous cAMP in both strain backgrounds but showed much lower activity in PA14 than in PA103 regardless of media conditions (Fig. 4).

*P. aeruginosa* cAMP levels are controlled by two adenylate cyclases, CyaA and CyaB, and the phosphodiesterase CpdA (37, 42). Expression of CpdA from a plasmid (pCpdA) reduced expression of the P*_lacP1_* cAMP reporter in PA103 bacteria; expression of the *attB::P_exoT_* T3SS-reporter was likewise reduced (Fig. 5A and B). Although no further reduction of the already low levels of P*_lacP1_* reporter expression was seen in PA14 carrying pCpdA (Fig. 5A), the introduction of this plasmid into the dual reporter strain eliminated both primed cells (Fig. 5C) and RFP+/GFP+ T3SS-ON cells (Fig. 5D).

**Fig 6:**
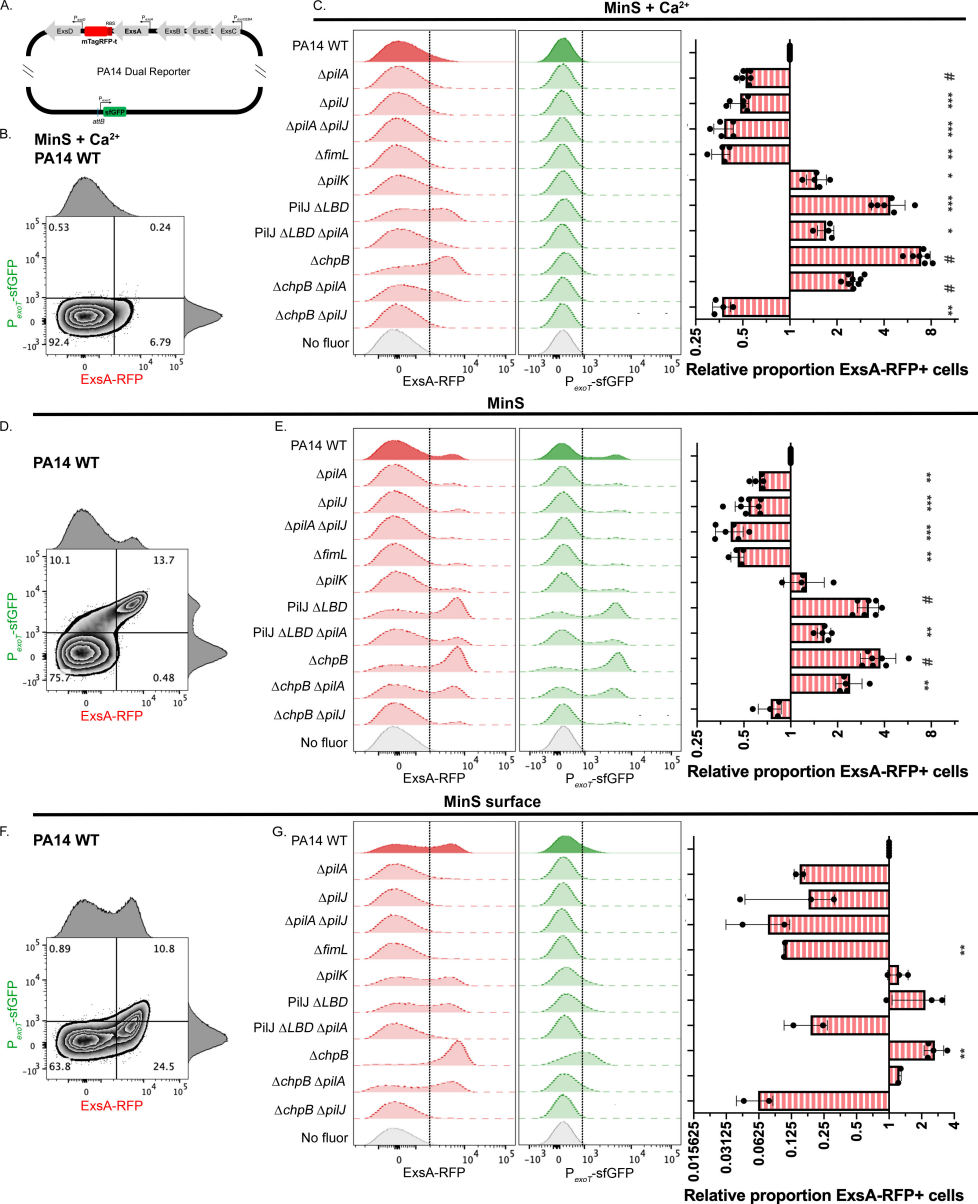
Mutation of Pil/Chp genes affects T3SS-priming. (**A**) All mutants were constructed in the PA14 dual reporter construct background; diagram replicated from Fig. 1A. Wild-type and mutant bacteria were grown planktonically for 7 h in MinS + Ca^2+^ (**B and C**) or MinS (**D and E**), or plated to MinS agar for 7 h (**F and G**) prior to sampling and flow cytometry. Representative flow cytometry zebra plots for the wild-type strain grown in each condition (**B, D, and F**) also show adjunct histograms of fluorescence intensity for ExsA-RFP and P*_exoT_*-sfGFP; such representative fluorescence intensity histograms are plotted separately for wild-type and mutant strains (**C, E, and G**) to facilitate interstrain comparisons. For all analyses, quadrants were established on a WT PA14 no-fluor control and validated on a 1:1 mixture of single GFP+ and RFP+ PA14 samples. The PA14 no-fluor control (gray histogram) is included to show the position of RFP and GFP gates (dotted line) for panels C, E, and G. Data collected across multiple flow cytometry experiments were normalized as follows and are displayed in the adjoining bar graphs (**C, E, and G**). For each experiment, the percentage of ExsA+/RFP+ bacteria in a sample was normalized to the percentage of ExsA+/RFP+ wild-type dual reporter cells (run as a positive control within every experiment), allowing the relative change in ExsA+/RFP+ percentage associated with T4P mutations to be calculated. Bar graphs show geometric mean fold-change in the ExsA+/RFP+ population ± geometric SD for every mutant relative to wild type. Each point represents a biological replicate (*n* = 2–8). Significance was determined by one-way ANOVA with Dunnett’s multiple comparisons test compared to WT. Adjusted *P*-values: *, <0.05; **, <0.01; ***, <0.001; #, <0.0001. Comparisons that did not achieve statistical significance (*P* > 0.05) are unmarked.

Adenylate cyclase activity is positively regulated by the Pil/Chp chemosensory pathway (41, 43, 44). The PilJ chemoreceptor signal and/or ligand(s) have largely been studied in surface-associated bacteria. T4P retraction by PilT, PilA interaction with PilJ, and ligand sensing by PilJ have all been identified as cues for Pil/Chp activation, with FimL serving as a required polar scaffold (43–48). We introduced unmarked deletions of *pilA*, *pilJ, fimL*, and *pilA pilJ* into the PA14 dual reporter background—all expected to disrupt Pil/Chp pathway activity—and tested whether they altered the proportion of T3SS-primed bacteria grown planktonically. Each mutation consistently reduced the proportion of ExsA+/RFP+ cells in the absence (MinS + Ca^2+^) (Fig. 6A through C) or presence (MinS) (Fig. 6D and E) of a T3SS-activating signal. These phenotypes could be complemented by expressing the deleted gene *in trans* (Fig. S7). Cells were also cultured for the same time on MinS agar plates, then scraped and subjected to flow cytometry; here, the loss of *pilA*, *pilJ,* or *fimL* led to a more pronounced decrease in the proportion of ExsA+/RFP+ primed cells relative to wild-type populations (Fig. 6F and G).

The activity of the PilJ chemoreceptor is modulated by the methylesterase ChpB and the methyltransferase PilK, whose disruption increases or decreases cAMP in surface-grown bacteria, respectively (49). Deletion of *chpB* in the PA14 dual reporter background markedly increased the proportion of primed cells under both planktonic and surface growth conditions; surprisingly, deletion of *pilK* was also associated with a slight increase in primed cells (Fig. 6C, E, and G). PilJ possesses a periplasmic ligand-binding domain (LBD) that responds to signals that are still incompletely characterized (46). Deletion of this domain (aa 39–303) in chromosomally encoded PilJ (PilJ∆LBD) was also sufficient to increase proportions of primed cells after planktonic or surface growth (Fig. 6B through G). The positive effect of ∆*chpB* on priming disappeared when *pilJ* was also deleted (∆*chpB* ∆*pilJ*), consistent with ChpB’s role as a PilJ methylesterase (Fig. 6C, E, and G). We also deleted *pilA* in combination with ∆*chpB* or PilJ∆LBD. When planktonically grown, these double mutants had reduced populations of ExsA+/RFP+ cells as compared to the single ∆*chpB* or PilJ∆LBD strains; however, primed populations remained higher than observed for ∆*pilA* bacteria (Fig. 6B through E). This may indicate that the PilA-PilJ interaction makes a lesser contribution to Pil/Chp activation in planktonically grown cells, especially when PilJ is hyperactivated. In contrast, deletion of *pilA* in the PilJ∆LBD background reduced the primed ExsA+/RFP+ population to levels seen for ∆*pilA* when bacteria were surface grown (Fig. 6G). This might reflect a greater role for Type 4 pilus dynamics in activating Pil/Chp in surface-grown cells.

In aggregate, our data suggest that mutations in the Pil/Chp system affect the phenotypes of priming and T3SS via their effects on endogenous cAMP levels. In keeping with this, Pil/Chp mutations associated with decreased priming could be complemented by providing exogenous cAMP (Fig. S8). We also introduced the *attB*::P*_lacP1_*-sfGFP reporter to assess cAMP in the PilJ∆LBD background; compared to its isogenic parent, activity of the cAMP reporter was modestly but reproducibly increased by the PilJ∆LBD allele (Fig. S9). The association of a small change in *lacP1*-sfGFP fluorescence with large changes in ExsA and P*_exoT_* reporter activity is consistent with cAMP serving as a signal that “flips the switch” on a positive feedback circuit that pushes cells into an ExsA-expressing, primed state (50).

## DISCUSSION

Heterogeneous expression of Type III secretion genes within clonal populations is broadly observed. Such cooperative virulence—in which T3SS-ON individuals incur a measurable fitness cost to produce a virulence factor with shared benefit—allows genetically identical T3SS-OFF individuals to survive within a host without loss of the T3SS-positive genotype (7, 23, 51). In *P. aeruginosa*, time-lapse microscopy has established that T3SS-ON cells arise from a subpopulation of primed cells, which express the transcriptional activator ExsA in the absence of T3SS-activating signals (26). When exposed to activating signals, for example, divalent chelators such as NTA, these ExsA+ cells immediately respond by expressing T3SS effector genes. We also observed cells that became ExsA+ while growing in the presence of activating signals; after correcting for fluorescence protein maturation time, an average of 90 min elapsed before they also began expressing the T3SS effector reporter (26). We wondered whether this lag represented the time required to express and assemble an injectisome capable of responding to T3SS-activating signals.

ExsE secretion through the T3SS needle links T3SS activation with the induction of ExsA-dependent effector expression by triggering a partner-switching cascade, which sequesters the anti-activator ExsD and frees ExsA to homodimerize and bind DNA (33–35). The partner-switching model predicts that primed cells carry T3SS injectisomes, which we demonstrated in this study using whole-cell cryo-ET. ExsA is encoded within the ExsA-activated *exsCEBA* operon, and thus subject to positive transcriptional feedback; its anti-activator, ExsD, is likewise encoded within an ExsA-activated *exsDpscBCDEFGHIJKL* operon, which contains many of the T3SS injectisome structural genes. ExsA therefore positively regulates the expression of its anti-activator, ExsD, as well as that of the anti-anti-activator ExsC and its binding partner ExsE. However, Yahr and colleagues discovered a weak promoter upstream of *exsA* (P*_exsA_*), which is activated by cAMP-bound Vfr and transcribes only *exsA* (25). Working in PA103 under T3SS-activating conditions, they showed that deletion of *vfr* decreased the bistable proportion of cells expressing transcriptional reporters for either P*_exsD_* or P*_exoS_*, while disruption of the P*_exsA_* promoter itself completely ablated expression of either reporter. Based on their observations, they proposed that this Vfr-dependent stimulation of *exsA* expression could “alter the stochastic balance of the system,” and invoked earlier studies positively correlating proportions of T3SS-expressing cells with metabolic status and intracellular cAMP levels (25).

In this work, whole-cell cryo-ET was used to test whether bacteria assemble injectisomes in the absence of T3SS-activating signals. This was clearly the case, with the proportion of PA14 cells assembling T3SS needles (14%) similar to that scored as ExsA+/RFP+ by microscopy (10%) in the dual-reporter strain, where we could employ longer exposures to capture signals from this chromosomal reporter (26). The challenge of detecting these dim ExsA+/RFP+ cells by flow cytometry (~5%) likely contributed to the discrepancy seen here, as the ExsA+/T3SS-ON population that arose from cells growing in MinS (where positive feedback amplified the ExsA+/RFP+ signal; Fig. 1D) was always larger than the corresponding RFP+ population estimated in MinS + Ca^2+^ (Fig. 1C vs B). When cells grown in activating conditions were examined, about 17% had injectisomes, while 20%–25% were ExsA+/T3SS-ON by flow cytometry (Fig. 2). The similar proportions of injectisome-bearing cells before and after activation was not surprising to us, as an activating signal would be perceived by cells with injectisomes and induce expression of ExsA-dependent genes within that population.

We next tested whether exogenous cAMP would increase the proportion of primed cells, that is, those that expressed ExsA and assembled injectisomes in the absence of activating signals, and responded to activating signals with P*_exoT_*-GFP expression. This was indeed the case. Although we could not see both ExsA expression (fluorescence) and injectisome assembly (whole-cell cryo-ET) in the same cells over time, the proportions of cells expressing these traits again tracked closely (Fig. 3). Whole-cell cryo-ET did not have sufficient resolution to confirm that cytosolic components were associated with needles/basal bodies; however, interrogation of *lux*-based reporters for each T3SS operon showed that all of those encoding proteins associated with the T3SS injectisome were transcribed in these calcium-replete conditions, unlike effectors (Fig. S6). The addition of cAMP increased the expression of these structural operons. In a ∆*exsA* mutant background, however, expression with or without cAMP was comparable to that of a promoterless *lux* reporter (P*_null_*).

**Fig 7:**
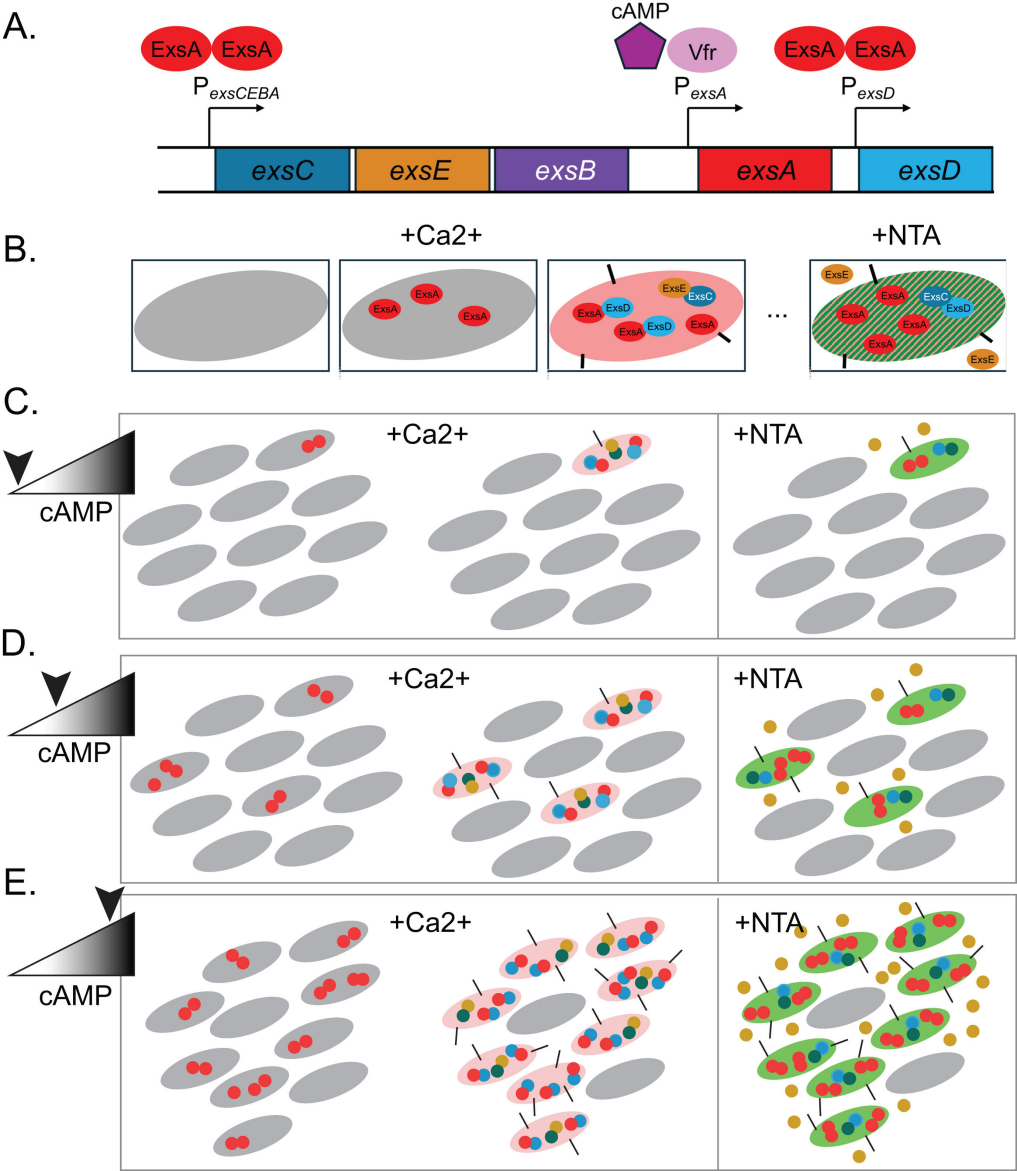
The cAMP-responsive *P_exsA_* promoter coupled with positive feedback at *P_exsCEBA_* creates a bistable distribution of primed cells. (**A**) T3SS regulatory genes schematic, including ExsA-dependent (*P_exsCEBA_*, *P_exsD_*) and ExsA-independent (*P_exsA_*) promoters. (**B**) In calcium-replete (non-activating) conditions, transcriptional activity at the P*_exsA_* promoter can lead to ExsA production, resulting in ExsA-dependent expression of T3SS regulatory and structural genes. Assembled T3SS injectisomes persist even after ExsA-ExsD and ExsC-ExsE heterodimers form and inhibit further T3SS gene expression. An activating signal (e.g., the calcium chelator NTA) triggers ExsE secretion, resulting in ExsC-ExsD heterodimer formation and resumption of ExsA-dependent gene expression. (**C**) When intracellular cAMP is low, the *P_exsA_* promoter fires infrequently, and few cells become primed. (**D**) When intracellular cAMP levels are higher, transcriptional activity from *P_exsA_* increases, raising the probability that a cell will express ExsA and become primed. (**E**) Treatment with cAMP drives transcription from *P_exsA_* in most cells, leading to a high percentage of primed bacteria. (Red circles, ExsA; blue circles, ExsD; teal circles, ExsC; gold circles, ExsE.)

The sequestration-based ExsA/D/C/E regulatory circuit provides an ultrasensitive mechanism for generating bistability, as has been demonstrated by reconstituting this signaling cascade in *E. coli* (52). Although noisy transcription from the P*_exsA_* promoter can drive a cell to produce enough ExsA to establish a T3SS needle-expressing, primed state—per observations that some T3SS expression occurs even in the absence of Vfr (∆*vfr*) or adenylate cyclases (∆*cyaA*∆*cyaB*), but not if the P*_exsA_* promoter is disrupted (25)—high levels of cAMP shifted the entire population to this needle-expressing, primed phenotype (Fig. 3). We also observed that mutations predicted to increase or decrease intracellular cAMP levels increased or decreased, respectively, the percentage of primed cells within otherwise isogenic populations (Fig. 5 and 6). This is consistent with Marsden et al.’s proposal that cAMP-Vfr-dependent expression of *exsA* would “alter the stochastic balance of the system” between cells that do and don’t express T3SS (25). It may seem surprising that small changes in cAMP (as detected with the P*_lacP1_* transcriptional reporter) led to large changes in primed population size, but this behavior is predicted when a signal (i.e., cAMP) increases the rate of *exsA* expression enough to establish an “on-state” (ExsA produced) that is stabilized by ExsA’s positive feedback on its own expression from P*_exsCEBA_* (Fig. 7) (50). This primed phenotype can then persist—even if cAMP levels fall below the threshold required for *exsA* expression—until a T3SS-activating signal (e.g., NTA chelation of calcium) triggers ExsE secretion, ExsC-ExsD binding, and ExsA-dependent transcription of T3SS effectors. For a strain like PA103, where endogenous intracellular cAMP levels appear very high (Fig. 4), most cells seem to express enough *exsA* to become primed, as seen by the very high proportion of bacteria with one or more injectisomes (Fig. 2).

cAMP production is mechanistically linked to T4P assembly/retraction via the Pil/Chp chemotaxis system, which activates the CyaB adenylate cyclase (41). Surface-attached *P. aeruginosa* increases cAMP production in a T4P-dependent manner (43, 53). Coupling T3SS expression to surface attachment could provide *P. aeruginosa* with a defense mechanism against eukaryotic predators when it is at a surface but not yet within a biofilm community (41, 54). Most of our experiments were carried out with planktonically cultured bacteria to create a uniform environment for examining phenotypic heterogeneity. Even so, we observed that mutations that activated the PilJ chemoreceptor of the Pil/Chp system increased the percentage of primed bacteria, while deletion of this signaling system decreased the population of primed cells (Fig. 6). When the dual reporter strain was cultured on MinS agar, the percentage of ExsA+/RFP+ primed cells was consistently larger than in MinS liquid (Fig. 6D vs F), consistent with increased cAMP levels in surface-associated bacteria. Pil/Chp-disrupting mutations also had larger effects on primed population size in these surface-grown cells, as might be expected given the role of Type 4 pili in cAMP production.

Though T3SS regulation differs among pathogens, many bacteria exhibit bimodal T3SS expression (7, 55). In *Salmonella* Typhimurium, T3SS-1 expression is controlled by a complex “feed-forward” loop of multiple positive autoregulatory elements. The strongest, HilD, directly promotes the expression of HilA, the T3SS-1 transcriptional activator, while two other positive autoregulators (HilC and RtsA) serve as rheostatic amplifiers for HilA (56). HilD is repressed by binding to HilE, the main negative T3SS-1 regulator; this interaction primarily controls the bistable switch and sets the threshold for generating a T3SS-1 “ON” cell (56, 57). Unlike *P. aeruginosa’s* ExsD, HilE expression is not controlled by HilA but rather by PhoPQ (58). *S.* Typhimurium T3SS-1 expression is bistable, though hypoxia—which inactivates HilE—can cause a shift to unimodal T3SS-1+ expression (59). As additional transcriptional activators for the T3SS-1 are downstream of HilA, the *S.* Typhimurium system acts in a multi-step, hierarchical process with the potential to create primed cells (56). Small increases in HilD, combined with inputs from HilC and RtsA, could shift the balance between HilA and HilE, resulting in the expression of T3SS structural genes before cells receive the T3SS-activating signal of host-cell contact (56). Heterogeneous T3SS expression is not limited to mammalian pathogens but is also found in plant pathogens, including *Dickeya dadantii* and *P. syringae*, suggesting that T3SS priming could be more widespread if it is a key feature of bistability (60, 61).

To our knowledge, no single-cell studies of T3SS expression have been carried out in *Vibrio* spp*.* to determine whether T3SS expression is bimodal. *V. alginolyticus,* an opportunistic marine animal and human pathogen, does utilize a partner-switching mechanism to control T3SS gene expression and requires ExsE secretion for T3SS activation and upregulation (62). This requirement for ExsE secretion could suggest that T3SS needles are assembled prior to receiving a secretion signal, similar to our observations in *P. aeruginosa*. The related species, *V. parahaemolyticus,* also regulates its T3SS1 through partner-switching but is not reliant on ExsE secretion to increase T3SS-mediated virulence (33, 62–66). Crucially, ExsA does not regulate its own expression in either of these species, eliminating one known mechanism for establishing bistability (67).

In conclusion, T3SS are critical virulence factors whose expression carries significant fitness costs for individual bacteria. In *P. aeruginosa*, the ability to express the transcriptional activator ExsA independently of ExsD, ExsC, and ExsE from the P*_exsA_* promoter is required to establish bimodal T3SS expression: deletion of Vfr markedly reduces the proportion of bacteria expressing T3SS genes, while disruption of the P*_exsA_* promoter itself completely ablates T3SS expression (25, 68). Basal levels of transcription from this P*_exsA_* promoter lead to small populations of primed bacteria ready to respond to T3SS-activating signals, while cAMP-dependent transcription allows the proportion of primed bacteria to change in response to environmental and metabolic cues that modulate this second messenger.

## MATERIALS AND METHODS

### Bacterial growth

Bacterial strains and plasmids are listed in Table S1. Bacteria were grown at 37°C with aeration (250 rpm) in Miller’s Luria broth (LB) (10 g/L casein digest peptone, 10 g/L sodium chloride, and 5 g/L yeast extract) unless otherwise indicated. For biparental matings, *P. aeruginosa* was selected on Vogel-Bonner medium (VBM) agar (69). When necessary, antibiotics were added as follows: for *E. coli*, 15 μg/mL gentamicin, 20 μg/mL tetracycline, 100 µg/mL ampicillin; for *P. aeruginosa*, 30 µg/mL (CRISPR mating, *attTn7* complementation) or 100 µg/mL gentamicin, 100 µg/mL tetracycline, 200 µg/mL carbenicillin.

To activate T3SS gene expression, *P. aeruginosa* was grown in MinS medium; 10 mM nitrilotriacetic acid (NTA) was added to MinS when indicated (MinS + NTA) (26). To repress T3SS gene expression, *P. aeruginosa* was grown in MinS medium plus 5 mM CaCl_2_ (MinS + Ca^2+^) (70). As indicated, cyclic-AMP (cAMP) (MedChemExpress) dissolved in 50 mM Tris-HCl (pH = 8) was added at a final concentration of 20 mM.

### Plasmid and strain construction

Reporters for ExsA (*exsA*-IRES-mTagRFP-t) and T3SS gene expression (*attB*::P*_exoT_*-sfGFP) were previously constructed and integrated into PA14 (26). To report relative cAMP levels, the P*_lacP1_*-sfGFP reporter was integrated at the *attB* site (41, 71). Biparental mating was carried out on low-salt LB agar plates (5 g/liter NaCl), and integrants were selected on VBM-tetracycline plates. The vector backbone was excised by mating with *E. coli* SM10 pFLP2 (72). Loss of vector backbone and pFLP2 curing was confirmed by PCR for tetracycline and carbenicillin-sensitive colonies. The *pilJ* gene was deleted in the PA14 dual reporter by homologous recombination, using a Gateway-adapted pDONRx plasmid containing ~1 kb upstream and downstream flanks for *pilJ*, and merodiploids selected on VBM-gentamicin, followed by counterselection on VBM plus 15% (wt/vol) sucrose to promote backbone loss (41). ∆*pilA*, *∆chpB, ∆chpB ∆pilA, ∆chpB ∆pilJ, ∆pilK*, *∆fimL*, *pilJ* ∆LBD1-2, and *pilJ* ∆LBD1-2 ∆*pilA* constructs were generated using the CRISPR-Cas9 system (73). PAM sites and single-guide RNA sequences were identified using Geneious Prime 2025.2. (http://www.geneious.com). pS648 plasmids with the target sequence and pSH124-*ssr* were electroporated into the PA14 dual reporter and selected on LB plates containing carbenicillin, gentamicin, and 5 mM m-toluic acid. T4P deletion mutants were complemented chromosomally at the *att-*Tn7 insertion site via biparental mating of PA14 ∆T4P *attB::*P*_exoT_-sfGFP exsA-*IRES-*mTagRFP-t* with pUC18T-*T4P*, and helper conjugation plasmids pTSN2 and pRK2013 (74, 75). The pUC18T*-T4P* constructs were generated through PCR amplification of WT PA14 T4P sequences, including the native ribosome binding site as well as homologous overhangs to pUC18T, then assembled using HiFi Assembly (NEB) on a SacI/HindIII digested pUC18T. All constructs and deletions were validated by Sanger sequencing (Keck Sequencing Facility, Yale). Primers for this study, including spacer guide sequences, are listed in Table S2.

### Flow cytometry

Single colonies were inoculated into LB and grown overnight with aeration at 37°C, subcultured into LB and grown until early log-phase, washed with MinS, and diluted to a starting OD_600_ of 0.01 into the indicated MinS medium. For surface-grown samples, bacteria were spread at an OD_600_ of 0.01 on 25 mL MinS agar plates. Cultures were grown for a further 4–7 h before sampling and flow cytometry. For surface experiments, the entire agar plate was scraped and resuspended in PBS-MC (phosphate-buffered saline, pH 7.4, plus 0.9 mM calcium, 0.9 M magnesium), then processed for flow cytometry (76). Briefly, bacterial cells were fixed with 1% paraformaldehyde with mixing at 37°C for 30 min, diluted to an OD_600_ of 0.04 in PBS-MC, then passed through a 40 µm filter. Cells were analyzed for GFP emission in B525-FITC with a 525/40 bandpass filter and RFP emission in 584-mCherry with a 584/42 bandpass filter using a CytoFlex LX equipped with a 96-well plate sampler (Beckman Coulter). At least 50,000 events were collected per sample. Isogenic WT *P. aeruginosa* bacteria lacking fluorescent reporters were used as gating controls as they showed equal fluorescence to the PA14 ∆*exsA* P*_exoT_*-sfGFP (Fig. S2). The full gating scheme is detailed in Fig. S1.

Flow cytometry data were analyzed using FlowJo v10 software. Zebra plots were used to simultaneously display RFP and GFP fluorescence, with the “adjunct histogram” feature enabled to show the relationship between these contour plots and the individual RFP and GFP histograms used to facilitate visualization of interstrain comparisons (Fig. S3).

### Cryo-electron tomography sample preparation and data analyses

*P. aeruginosa* strains PA103 and PA14 were grown as described above, either in MinS + 5 mM Ca^2+^ or MinS + NTA media as indicated, with either 20 mM cAMP or Dulbecco-PBS (D-PBS) added. After growth, bacterial concentrations were adjusted to OD_600_ 1.0 in DPBS. Bacterial samples and BSA-coated gold tracer (10 nm) solution (Aurion) were then mixed at a ratio of 1:1 (vol/vol). Five microliters of the mixture was deposited onto freshly glow-discharged cryo-EM grids (Quantifoil, Cu, R2/1, 200 mesh). Filter paper (Whatman) was used to blot the sample for ~5 s from the back of the cryo-EM grid, which was then immediately plunged into a liquid ethane and propane mixture using a homemade gravity plunger. Frozen-hydrated specimens of *P. aeruginosa* were imaged at ~−180°C using a Titan Krios G2 300 kV transmission electron microscope (Thermo Fisher Scientific) equipped with a field emission gun and a K3 direct detection camera with a GIF BioQuantum Imaging Filter (Gatan). SerialEM software and FastTomo script were used to record tilt series images for each bacterial target at a magnification of 15,000 (77, 78). The physical pixel size at the specimen level is 6.184 Å. The angle of the tilt series ranged between ±48° in 3° increments. The total electron dose was ~60e^−^/Å^2^ distributed across 33 images of each tilt series. The defocus was set at −10 μm without a volta phase plate and −1.0 μm with a phase plate (Table S3). MotionCor2 was used to correct image drift during data collection (79). Drift-corrected images were combined to create image stacks for each tilt series by IMOD (80). Fiducial beads in the images were used to align a tilt series by IMOD (80). The binvol function in IMOD was used to generate 4× binning of the aligned stacks, and 4× binned tomograms with SIRT reconstruction were then reconstructed using Tomo3D (81, 82). The number of tomograms for each specimen is shown in Table S3. Injectisomes on the bacterial surface were counted manually. Surface rendered tomogram images were generated using Dragonfly software (version 2022.2, Comet Technologies Canada). For subtomogram averaging, 373 injectisomes were selected from the 4× binned tomograms. From these, 346 unbinned subtomograms reconstructed by weighted back projection (WBP) were extracted, and the binvol function in IMOD was used to generate 2× binned subtomograms for averaging. The averaged injectisome structure was determined by alignment of the complex using i3 software (83, 84).

### Statistical analysis

GraphPad Prism v10.5.0 was used for statistical analysis. Two-way comparisons were performed using two-tailed unpaired *t* tests with Welch’s correction to compare means for normally distributed data sets without assuming populations have equal variance. Multiple comparisons for normally distributed data were carried out using one-way ANOVA with Dunnett’s multiple comparisons test.

## SUPPLEMENTAL MATERIAL

Supplemental Material mbio.00545-26-s0001.pdf Supplemental methods, tables, and figures.

ASM does not own the copyrights to Supplemental Material that may be linked to, or accessed through, an article. The authors have granted ASM a non-exclusive, world-wide license to publish the Supplemental Material files. Please contact the corresponding author directly for reuse.
