# Supplementary material for: Injectisome assembly primes Pseudomonas aeruginosa for type III secretion
Source: mBio. 2026 Apr 30;17(6):e00545-26. doi: 10.1128/mbio.00545-26 (PMC13251382; doi:10.1128/mbio.00545-26)
Supplement: Supplemental Material — Supplemental methods, tables, and figures. [file mbio.00545-26-s0001.pdf]

Supplemental Materials for

Injectisome assembly primes *Pseudomonas aeruginosa* for Type III secretion

Kristen Ramsey[1,2], Shoichi Tachiyama[2, 3], Apolline Brossard[4], Zhao Hang[2, 3], Jun Liu[2, 3], Barbara I. Kazmierczak[2,5,#]

[1] Program in Microbiology, Yale University, New Haven, Connecticut, USA

[2] Department of Microbial Pathogenesis, Yale University, New Haven, Connecticut, USA

[3] Microbial Sciences Institute, Yale University, West Haven, Connecticut, USA

[4] Department of Molecular, Cellular and Developmental Biology, Yale College, New Haven, Connecticut

[5] Department of Medicine, Yale University, New Haven, Connecticut, USA

Supplemental Tables

Table S1. Bacterial strains and plasmids used in this study

**Strain or plasmid**	**Description or relevant genotype**	**Source or reference**
E. coli strains		
DH5α	*F- Φ80lacZΔM15 Δ(lacZYA-argF) U169 recA1 endA1 hsdR17 (rk-, mk+) phoA supE44 λ-thi-1 gyrA96 relA1.* Used for cloning.	Invitrogen DH5α max efficiency competent cells
S17.1	*recA pro hsdR* RP4-2-Tc::Mu-Km::Tn7 λpir Used for mating constructs into *P. aeruginosa*	(1)
P. aeruginosa strains		
PA14	Wild type isolate	F. Ausubel
PA14 *attB*::P_{exoT}-*sfGFP exsA*-RBS-*mTagRFP-t*	*sfGFP* under the control of native *exoT* promoter integrated in *attB* site*; mTagRFP-t* gene inserted after *exsA* gene with its own RBS, 'dual reporter'	(2)
PA14 *attB*::P_{exoT}-*sfGFP exsA*-RBS-*mTagRFP-t* pMQ72-*cpdA*	Dual ExsA/T3SS reporter expressing a plasmid with arabinose-inducible cAMP phosphodiesterase *cpdA;* Gm^R	This work
PA14 *attB*::P_{exoT}-*sfGFP exsA*-RBS-*mTagRFP-t* pMQ72	Dual ExsA/T3SS reporter with empty vector control for CpdA experiments*;* Gm^R	This work
PA14 *attB*::lacP1-*sfGFP*	*sfGFP* under the control of cAMP-responsive *lacP1* promoter integrated at the *attB* site	This work
PA14 Δ*pilA attB::*P_{exoT}-*sfGFP exsA*-IRES-*mTagRFP-t*	Deletion of *pilA* in Dual ExsA/T3SS reporter background	This work
PA14 Δ*pilA attB::*P_{exoT}-*sfGFP exsA*-IRES-*mTagRFP-t att*-tn7*::*p_{tac}-*pilA*	Deletion of *pilA* in Dual ExsA/T3SS reporter background; *pilA* complement inserted under control of IPTG-inducible *tac* promoter in *att*-Tn7 site	This work
PA14 Δ*pilJ attB::*P_{exoT}-*sfGFP exsA*-IRES-*mTagRFP-t*	Deletion of *pilJ* in Dual ExsA/T3SS reporter background	This work
PA14 Δ*pilJ attB::*P_{exoT}-*sfGFP exsA*-IRES-*mTagRFP-t att*-tn7*::*p_{tac}-*pilJ*	Deletion of *pilJ* in Dual ExsA/T3SS reporter background; *pilJ* complement inserted under control of IPTG-inducible *tac* promoter in *att*-Tn7 site	This work
PA14 Δ*pilA* Δ*pilJ attB*::P_{exoT}-*sfGFP exsA*-IRES-*mTagRFP-t*	Deletion of *pilA* and *pilJ* in Dual ExsA/T3SS reporter background	This work
PA14 Δ*pilA* Δ*pilJ attB::*P_{exoT}-*sfGFP exsA*-IRES-*mTagRFP-t att*-tn7*::*p_{tac}-*pilA*	Deletion of *pilA* and *pilJ* in Dual ExsA/T3SS reporter background; *pilA* complement inserted under control of IPTG-inducible *tac* promoter in *att*-Tn7 site	This work
PA14 Δ*pilA* Δ*pilJ attB::*P_{exoT}-*sfGFP exsA*-IRES-*mTagRFP-t att*-tn7*::*p_{tac}-*pilJ*	Deletion of *pilA* and *pilJ* in Dual ExsA/T3SS reporter background; *pilJ* complement inserted under control of IPTG-inducible *tac* promoter in *att*-Tn7 site	This work
PA14 PilJ ΔLBD *attB*::P_{exoT}-*sfGFP exsA*-IRES-*mTagRFP-t*	Deletion of *pilJ* ligand binding domain (amino acids 39 – 303); in Dual ExsA/T3SS reporter background	This work

PA14 PilJ ΔLBD Δ*pilA* *attB*::P$_{exoT}$-*sfGFP exsA*-IRES-*mTagRFP-t*	Deletion of *pilA* and PilJ ligand binding domain (amino acids 39 – 303) in Dual ExsA/T3SS reporter background	This work
PA14 PilJ ΔLBD Δ*pilA* *attB*::P$_{exoT}$-*sfGFP exsA*-IRES-*mTagRFP-t att*-tn7::p$_{tac}$-*pilA*	Deletion of *pilA* and PilJ ligand binding domain (amino acids 39 – 303) in Dual ExsA/T3SS reporter background; *pilA* complement inserted under control of IPTG-inducible *tac* promoter in *att*-Tn7 site	This work
PA14 Δ*pilK attB*::P$_{exoT}$-*sfGFP exsA*-IRES-*mTagRFP-t*	Deletion of *pilK* in Dual ExsA/T3SS reporter background	This work
PA14 Δ*chpB attB*::P$_{exoT}$-*sfGFP exsA*-IRES-*mTagRFP-t*	Deletion of *chpB* in Dual ExsA/T3SS reporter background	This work
PA14 Δ*chpB* Δ*pilJ* *attB*::P$_{exoT}$-*sfGFP exsA*-IRES-*mTagRFP-t*	Deletion of *pilJ* and *chpB* in Dual ExsA/T3SS reporter background	This work
PA14 Δ*chpB attB*::P$_{exoT}$-*sfGFP exsA*-IRES-*mTagRFP-t att*-tn7::p$_{tac}$-*chpB*	Deletion of *chpB* in Dual ExsA/T3SS reporter background; *chpB* complement inserted under control of IPTG-inducible *tac* promoter in *att*-Tn7 site	This work
PA14 Δ*chpB* Δ*pilA* *attB*::P$_{exoT}$-*sfGFP exsA*-IRES-*mTagRFP-t*	Deletion of *chpB* and *pilA* in Dual ExsA/T3SS reporter background	This work
PA14 Δ*chpB* Δ*pilA* *attB*::P$_{exoT}$-*sfGFP exsA*-IRES-*mTagRFP-t att*-tn7::p$_{tac}$-*chpB*	Deletion of *chpB* and *pilA* in Dual ExsA/T3SS reporter background; *chpB* complement inserted under control of IPTG-inducible *tac* promoter in *att*-Tn7 site	This work
PA14 Δ*chpB* Δ*pilA* *attB*::P$_{exoT}$-*sfGFP exsA*-IRES-*mTagRFP-t att*-tn7::p$_{tac}$-*pilA*	Deletion of *chpB* and *pilA* in Dual ExsA/T3SS reporter background; *pilA* complement inserted under control of IPTG-inducible *tac* promoter in *att*-Tn7 site	This work
PA14 Δ*fimL attB*::P$_{exoT}$-*sfGFP exsA*-IRES-*mTagRFP-t*	Deletion of *fimL* in Dual ExsA/T3SS reporter background	This work
PA14 PilJ ΔLBD *attB*::P$_{lacP1}$-*sfGFP*	Deletion of PilJ ligand binding domain (amino acids 39 – 303); *sfGFP* under the control of cAMP-responsive *lacP1* promoter integrated in *attB* site	This work
PA103	Wild type isolate	(3), from J. Engel
PA103 Δ*exsA*	Unmarked deletion of *exsA*(Δaa 56-261) in PA103	(4), from A. Hauser
PA103 *attB*::P$_{exoT}$-*sfGFP*	*sfGFP* under the control of native *exoT* promoter integrated in *attB* site	This work
PA103 *attB*:: P$_{lacP1}$-*sfGFP*	*sfGFP* under the control of cAMP-responsive *lacP1* promoter integrated in *attB* site	This work
PA103 *attB*::P$_{exoT}$-*sfGFP* pMQ72-*cpdA*	Expressing a plasmid with arabinose-inducible cAMP phosphodiesterase *cpdA*. *sfGFP* under the control of native *exoT* promoter integrated in *attB* site; Gm^R	This work
PA103 *attB*:: P$_{lacP1}$-*sfGFP* pMQ72-*cpdA*	Expressing a plasmid with arabinose-inducible cAMP phosphodiesterase *cpdA*. *sfGFP* under the control of cAMP-responsive *lacP1* promoter integrated in *attB* site; Gm^R	This work
PA103 *attB*::P$_{exoT}$-*sfGFP* pMQ72	Empty vector control for CpdA experiments. *sfGFP* under the control of native *exoT* promoter integrated in *attB* site; Gm^R	This work

PA103 *attB*:: P*lacP1*-*sfGFP* pMQ72	Empty vector control for CpdA experiments. *sfGFP* under the control of cAMP-responsive *lacP1* promoter integrated in *attB* site; Gm^R	This work
PA103 *attB*::Pnull-*luxCDABE*	Promoterless luciferase reporter in PA103	(5)
PA103 Δ*exsA* *attB*::Pnull-*luxCDABE*	Promoterless luciferase reporter in PA103 Δ*exsA*	(5)
PA103 *attB*::P*exsC*-*luxCDABE*	*exsC* operon luciferase transcriptional reporter in PA103	(5)
PA103 Δ*exsA* *attB*::P*exsC*-*luxCDABE*	*exsC* operon luciferase transcriptional reporter in PA103 Δ*exsA*	(5)
PA103 *attB*::P*exsD*-*luxCDABE*	*exsD* operon luciferase transcriptional reporter in PA103	(5)
PA103 Δ*exsA* *attB*::P*exsD*-*luxCDABE*	*exsD* operon luciferase transcriptional reporter in PA103 Δ*exsA*	(5)
PA103 *attB*::P*popN*-*luxCDABE*	*popN* operon luciferase transcriptional reporter in PA103	(5)
PA103 Δ*exsA* *attB*::P*popN*-*luxCDABE*	*popN* operon luciferase transcriptional reporter in PA103 Δ*exsA*	(5)
PA103 *attB*::P*pscN*-*luxCDABE*	*pscN* operon luciferase transcriptional reporter in PA103	(5)
PA103 Δ*exsA* *attB*::P*pscN*-*luxCDABE*	*pscN* operon luciferase transcriptional reporter in PA103 Δ*exsA*	(5)
PA103 *attB*::P*pcrG*-*luxCDABE*	*pcrG* operon luciferase transcriptional reporter in PA103	(5)
PA103 Δ*exsA* *attB*::P*pcrG*-*luxCDABE*	*pcrG* operon luciferase transcriptional reporter in PA103 Δ*exsA*	(5)
PA103 *attB*::P*exoT*-*luxCDABE*	*exoT* operon luciferase transcriptional reporter in PA103	(5)
PA103 Δ*exsA* *attB*::P*exoT*-*luxCDABE*	*exoT* operon luciferase transcriptional reporter in PA103 Δ*exsA*	(5)
PA103 *attB*::P*exoU*-*luxCDABE*	*exoU* operon luciferase transcriptional reporter in PA103	(5)
PA103 Δ*exsA* *attB*::P*exoU*-*luxCDABE*	*exoU* operon luciferase transcriptional reporter in PA103 Δ*exsA*	(5)
PA103 *attB*::P*cyaB*-*luxCDABE*	*cyaB* luciferase transcriptional reporter in PA103	(5)
PA103 Δ*exsA* *attB*::P*cyaB*-*luxCDABE*	*cyaB* luciferase transcriptional reporter in PA103 Δ*exsA*	(5)
PA103 *attB*::P*vfr*-*luxCDABE*	*vfr* luciferase transcriptional reporter in PA103	(5)
PA103 Δ*exsA* *attB*::P*vfr*-*luxCDABE*	*vfr* luciferase transcriptional reporter in PA103 Δ*exsA*	(5)
Plasmids		
pDONRX	Gateway-adapted suicide vector; Gm^R	(8)
pDONRX-*pilJ*	Gateway-adapted suicide vector to delete *pilJ*; Gm^R	This work
mini-CTX2 P*exoT*-*sfGFP*	Vector to integrate P*exoT*-*sfGFP* cassette into <u>*attB*</u> insertion site; Tc^R	(2)
mini-CTX2 *lacP1-sfGFP*	Vector to integrate *lacP1-sfGFP* cassette into <u>*attB*</u> insertion site; Tc^R	(8)
pFLP2	To flip out mini-CTX2 backbone; Ap/Cb^R	(9)
pMMB67EH	Single copy expression vector with IPTG-inducible *tac* promoter, lacIQ+; Ap/Cb^R	(10)

pMMB-*mTagRFP-t*	*mTagRFP*-t cloned under control of IPTG-inducible *tac* promoter; Ap/Cb^R	(2)
pMMB-*sfGFP*	*sfGFP* cloned under control of IPTG-inducible *tac* promoter; Ap/Cb^R	(2)
pSH124-*ssr*	P_{trc} IPTG inducible *ssr* CRISPR recombinase; Ap/Cb^R	(11)
pS648·CsR	Carrying *cas9* nuclease and CRISPR counterselection marker; Gm^R	(11)
ΔLBD-pS648·CsR	Carrying *cas9* nuclease, CRISPR spacer to cut PilJ ligand binding domain (aa 39-303); Gm^R	This work
Δ*pilA*-pS648·CsR	Carrying *cas9* nuclease, CRISPR spacer to cut *pilA*; Gm^R	This work
Δ*fimL*-pS648·CsR	Carrying *cas9* nuclease, CRISPR spacer to cut *fimL*; Gm^R	This work
Δ*chpB*-pS648·CsR	Carrying *cas9* nuclease, CRISPR spacer to cut *chpB*; Gm^R	This work
Δ*pilK*-pS648·CsR	Carrying *cas9* nuclease, CRISPR spacer to cut *pilK*; Gm^R	This work
pMQ72	Multicopy expression vector with arabinose inducible P_{BAD} promoter; Gm^R	(12)
pCpdA	*P. aeruginosa* cAMP phosphodiesterase with ATG start site under control of arabinose inducible P_{BAD} promoter, in pMQ72; Gm^R	This work
pUC18T-*pilJ*	Vector to integrate *pilJ* under control of IPTG-inducible *tac* promoter in *att*-Tn7 insertion site; Gm/Ap^R	This work
pUC18T-*pilA*	Vector to integrate *pilA* under control of IPTG-inducible *tac* promoter in *att*-Tn7 insertion site; Gm/Ap^R	This work
pUC18T-*chpB*	Vector to integrate *chpB* under control of IPTG-inducible *tac* promoter in *att*-Tn7 insertion site; Gm/Ap^R	This work
pUC18T	Vector for *att*-Tn7 insertion cloning in *P. aeruginosa*; Gm/Ap^R	(13)
pTNS2	Helper vector for *att*-Tn7 insertion cloning in *P. aeruginosa*; Ap^R	(13)
pRK2013	Helper vector used for *att*-Tn7 insertion mating in *P. aeruginosa*; Km^R	(14)

Table S2. Primers used in this study

Purpose	Primer Name	Details	Primer Sequence 5' → 3'
Construction of *pilJ* deletion mutant	PilJ Up F	Underline = site for Gateway cloning	GGGGACAAGTTTGTACAAAAAAGCAGGCTCCCGTCAGTTGACCAAGGAC
	PilJ Up R		CTATGCTCAGGCCTGCTCCAGGACCGACCACGATTACGA
	PilJ Down F	Underline = site for Gateway cloning	TCGTAATCGTGGTCGGTCCTGGAGCAGGCCTGAGCATAG
	PilJ Down R		GGGGACCACTTTGTACAAGAAAGCTGGGTACTCCATTCATGTGCCTGAG
To sequence pDONR constructs	attL1 F		CCAACTTTGTACAAAAAAGCAGGCT
	attL 1 R		CCAACTTTGTACAAGAAAGCTGGGT
To sequence pMQ72 constructs	pMQ72 F		CTCTCTACTGTTTCTCCATACC
	pMQ72 R		AGACCCCACACTACCATC
To isolate CpdA from PA14, mutate native TTG start site to ATG, and add overlap with pMQ72	CpdA F	Underline = pMQ72 homology, bold = change of T→A	TCTAGAGTCGACCTGCAGGCATGCAAGCTTCAGGAGACGGCCCC**A**TGTCACGCCATTCGA
	CpdA R	Underline = pMQ72 homology	GAAAATCTTCTCTCATCCGCCAAAACAGCCCATGTCAGTATCCGGCGGTGTCGTAGTCCA
To linearize pMQ72 with CpdA overlap	pMQ72 linear F	Underline = CpdA homology	TCGAATGGCGTGACAAGGGGCCGTCTCCTGAAGCTTGCATGCCTGCAGGTCGACTCTAGA
	pMQ72 linear R		TGGACTACGACACCGCCGGATACTGACATGGGCTGTTTTGGCGGATGAGAGAAGATTTTC
To sequence pUC18 constructs	Tn7 L	From (13)	AGCGGATAACAATTTCACACAGG
	Tn7 R		GCCCAAACATACAGGAAGAA
To sequence *P. aeruginosa att*-Tn7 insertions	GlmS Up	From (13)	CTGTGCGACTGCTGGAGCTGA
	GlmS Down		GCACATCGGCGACGTGCTCTC
To isolate *pilJ* with RBS from PA14 for *tn7* complementation	pUC-*pilJ* F	Underline = homology with pUC18T digested with SacI/HindIII	caatttcacacaggaaacagaattcgagcGCGGGGGCCAAATATGAAGAAAA
	pUC-*pilJ* R		ctgcaaggccttcgcgaggtaccgggcccaTATGCTCAGGCCTGCTCCACG
To isolate *pilA* with RBS from PA14 for *tn7* complementation	pUC-*pilA* F	Underline = homology with pUC18T digested with SacI/HindIII	caatttcacacaggaaacagaattcgagctATATCAATGGAGAGATACATGAAAG
	pUC-*pilA* R		ctgcaaggccttcgcgaggtaccgggcccaCTCTTTTCAGCATTAGCCTATTAGC
To isolate *chpB* with RBS from PA14 for *tn7* complementation	pUC-*chpB* F	Underline = homology with pUC18T digested with SacI/HindIII	caggaaacagaattcgagctAAGCCATCCAGTCCCTGG
	pUC-*chpB* R		ttcgcgaggtaccgggcccaTCATGTTTCGACTCCTGTCG
To confirm deletion of *pilJ* and PilJ ΔLBD	PilJ KO external F		GAAATCGTAATCGTGGTCGGT
	PilJ KO internal R		CTCGCCATCTTCGCCAGGT

To sequence Csr CRISPR spacer constructs	SEVA-T0-F	From (11)	GAACGCTCGGTTGCCGCC
To make PilJ ΔLBD CRISPR spacer	LBD 198 F	Used at 100 μM	GCGCGCGTACTGTCCCTGCACCAGG
	LBD 198 R		AAACCCTGGTGCAGGGACAGTACGC
Repair oligo to generate PilJ ΔLBD with CRISPR	PilJ ΔLBD Repair	Repair oligo, antisense strand	TGTTCATCGTCCTCATCGTCTCGATCGT GTTGCTGTTCGCCAACTTTGCCGGCGG GCGTAGCATCAACCTGTTCGCCGGTTAC GTCCTCGGCGCCCTGGC
To make Δ*pilA* with CRISPR	PilA 54 F	Used at 100 μM	CAGATAGGTTACCAGCGACA
	PilA 54 R		CAGATAGGTTACCAGCGACA
Repair oligo to generate Δ*pilA* with CRISPR	PilA Repair	Repair oligo, antisense strand	CATAAAAAAAGCCCCTCTTAGGAGGGGC TCTTTTCAGCATTAGCCTATTACATGTAT CTCTCCATTGATATATCCAGGCCTAACG CCTGACGATCATAAA
To confirm *pilA* deletion	PilA sequence F		CACTAACTCCATGACTGGCT
	PilA sequence R		CAGATAGGTTACCAGCGACA
To make Δ*fimL* with CRISPR	FimL 181 F	Used at 100 μM	GCGCGGCGCCCGCCTGGATATCCCG
	FimL 181 R		AAACCGGGATATCCAGGCGGGCGCC
Repair oligo to generate Δ*fimL* with CRISPR	FimL Repair	Repair oligo, antisense strand	CTGGGCGGTAGTCGGCCGACCTGCCTG CCAGCGGGACTCGCCGGCCATCACATC GGCCTAGTGCGCCTCCCGCGCAAGAAT ATACAACGGCATCAATCAG
To confirm *fimL* deletion	FimL sequence F		CGGGAGTTCCTGGCGCTTCA
	FimL sequence R		GTACGTCGCTACCTGGAACG
To make Δ*chpB* with CRISPR	ChpB 115 F	Used at 100 μM	GCGCGTTTCTCGACGCCTTGCCCGG
	ChpB 115 R		AAACCCGGGCAAGGCGTCGAGAAA C
Repair oligo to generate Δ*chpB* with CRISPR	ChpB Repair	Repair oligo, antisense strand	GCTCATGCCATCCTGCTCGATCACGGC CTGGTTCATGTTTCGACTCCTGTTCACT CATGCTGGCCGACCAGGGACTGGATGG CTTCGAGCAGCTCGGTTT
To confirm *chpB* deletion	ChpB sequence F		AACCAGTACCTCGGCAAGC
	ChpB sequence R		GGTTGCGATAGGCGATCAGT
To make Δ*pilK* with CRISPR	PilK 144 F	Used at 100 μM	GCGCGTCGAGCAGCTCGAAAGACGG
	PilK 144 R		AAACCCGTCTTTCGAGCTGCTCGAC
Repair oligo to generate Δ*pilK* with CRISPR	PilK Repair	Repair oligo, antisense strand	CCATTCCAGAGCGACGTAGTCGTGCCG GTCACCCATAGCCACTCCATTCACATGC CGTGCCCCTCGTCCAAGGTGTCGACGC GGCGCCCGCCAGGCGGCC
To confirm *pilK* deletion	PilK sequence F		TGGAGCAGGCCTGAGCATAG
	PilK sequence R		GTAGAACTCGACCATCTGCAGGG

Table S3. Cryo-ET tomogram collection.

Strains	Growth conditions	# of tomograms	# of injectisome for STA	Magnification (Å)	Vpp
PA103	MinS + 5mM Ca++	13	49	6.184	Yes
	MinS + 10mM NTA + cAMP	9	47	6.184	Yes
	MinS + 10mM NTA + PBS	13	23	6.184	Yes
PA14	MinS + 5mM Ca++ + cAMP	29	150	6.184	Yes
	MinS + 5mM Ca++	35	12	6.184	23 tomograms yes
	MinS + 10mM NTA + cAMP	11	86	6.184	Yes
	MinS + 10mM NTA + PBS	23	6	6.184	Yes
Total #	N/A	133	373	N/A	N/A

Supplemental Methods

In situ structure determination of P. aeruginosa injectisome by subtomogram averaging

Injectisomes in 4x binned tomograms with SIRT reconstruction were manually picked for the subtomogram averaging. Positions of the particles in 4x binned tomograms with weighted back projection (WBP) were aligned using i3 software to determine initial *in situ* structure of the injectisome (13). 2x binned subtomograms were used to refine and determine the *in situ* structure of the *P. aeruginosa* injectisome.

Western blotting for secreted T3SS proteins

T3SS components were detected by immunoblotting as previously described (14). Briefly, *P. aeruginosa* cultures were grown in 1mL of the indicated MinS minimal medium for 8 hours, then pelleted to separate secreted proteins from cells. Cell pellets were lysed by freeze-thaw at -80°C overnight, while the supernatant proteins were isolated by precipitation with 10%(v/v) trichloroacetic acid. Proteins were separated by SDS-PAGE using a 4-15% gradient and transferred to a PVDF membrane (Immobilon P, Millipore). Blots were blocked, then treated with anti-T3SS rabbit antiserum at a dilution of 1:20,000 in TBST plus milk (14). HRP-conjugated goat anti-rabbit IgG (Biorad) diluted 1:2,000 was used as secondary antibody. A Precision Plus Protein Dual Color Standards (Bio-Rad) ladder was used to estimate protein mass. Proteins were visualized by incubating membranes in 225 μM coumaric acid (Sigma), 1.25 mM 3-aminophthalhydrazide (Fluka) and 0.009% hydrogen peroxide (Fisher Scientific) in 100 mM Tris-HCl pH 8.5 for 1 min. Chemiluminescence was detected with a ChemiDoc MP imaging System using Image Lab Touch Software (Bio-Rad).

Transcriptional reporter luminescence assay

T3SS operon transcription was measured using PA103 and PA103 Δ*exsA* strains with the *luxCDABE* luciferase reporter fused to *exsCEBA* (P_{exsC}), *exsDpscBCDEFGHIJK* (P_{exsD}), *pscNOPQRTU* (P_{pscN}), *pcrGVHpopBD* (P_{pcrG}), *exoUspcU* (P_{exoU}), *popNpcr1234DR* (P_{popN}), *cyaB* (P_{cyaB}), *vfr* (P_{vfr}), or a promoterless control (P_{null}) integrated at the chromosomal *attB* site (5). All strains were inoculated from single bacterial colonies into LB and grown overnight with aeration at 37°C, subcultured into LB and grown until early log-phase, washed with MinS + Ca^{2+}, and diluted to a starting OD_{600} of 0.1 into MinS + Ca^{2+} supplemented with either 20 mM cAMP or a Tris-HCl vehicle control. Diluted strains were added to black-wall clear-bottom 96-well plates (Costar 3904) and incubated at 37°C with aeration in a plate-reader (Tecan Infinite M200pro) for 6 hours. Optical density (OD_{600}) was measured at 600 nm absorbance every 10 minutes and luminescence was also measured every 10 minutes throughout growth. Luminescence values are reported as relative luminescence units (RLU) normalized to OD_{600}.

Supplemental Figures

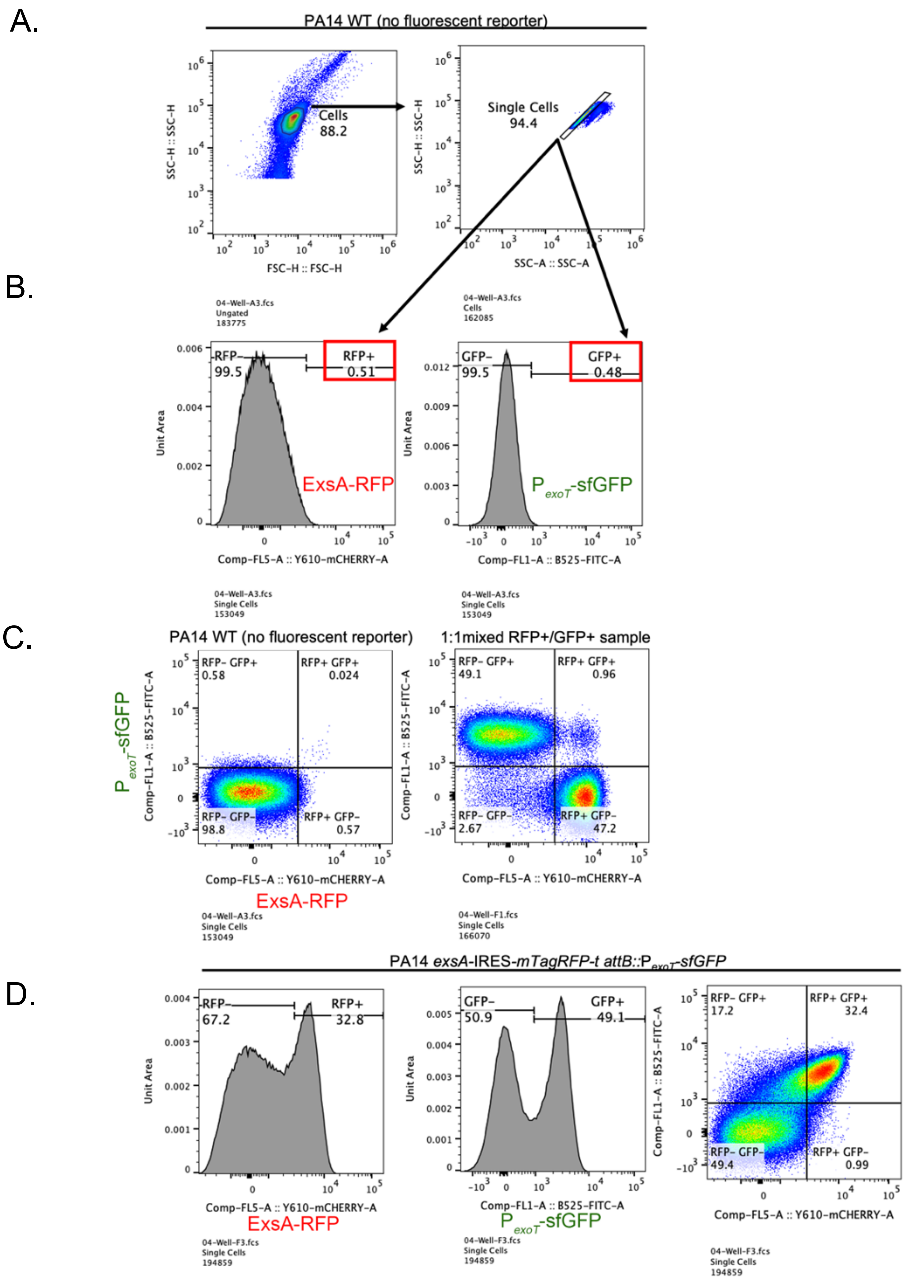

Figure S1. Flow cytometry gating scheme. (A) Cells were identified using a reporterless WT control by SSC-H vs FSC-H, then selectively gated for singlets under the assumption that SSC-H was approximately equal to SSC-A. (B) Within the 'single cells' gate, fluorescently positive cells were gated so as to exclude approximately 99.5% of the reporterless population. (C) To identify the remaining doublet error, RFP+/- and GFP+/- quadrants were established using the reporterless control, then applied to a 1:1 mix of constitutively RFP+ or GFP+ cells. Doublet error was consistently under 1%. (D) An example of the gating strategy applied to the PA14 dual reporter strain grown in MinS + NTA.

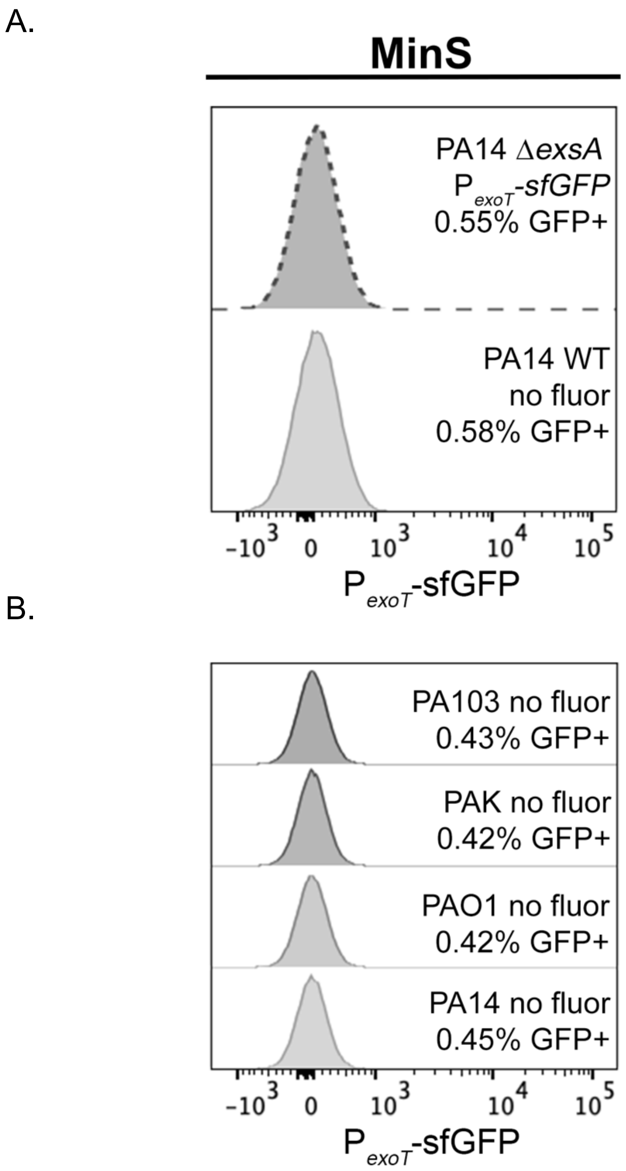

Figure S2. Establishment of negative controls for flow cytometry. (A) WT PA14 lacking a fluor and PA14 Δ*exsA* carrying the P_{exoT}-sfGFP T3SS reporter were grown in MinS for 7 hours, then analyzed for GFP fluorescence by flow cytometry. Fluorescent gates were established based on the no fluor *P. aeruginosa* PA14 WT control (solid grey). (B) Comparison across multiple *P. aeruginosa* WT strains lacking a fluorescent reporter to confirm that all strains have equivalent background fluorescence for drawing GFP gates. Fluorescent gates were established based on PA14 WT control (bottom, lightest grey).

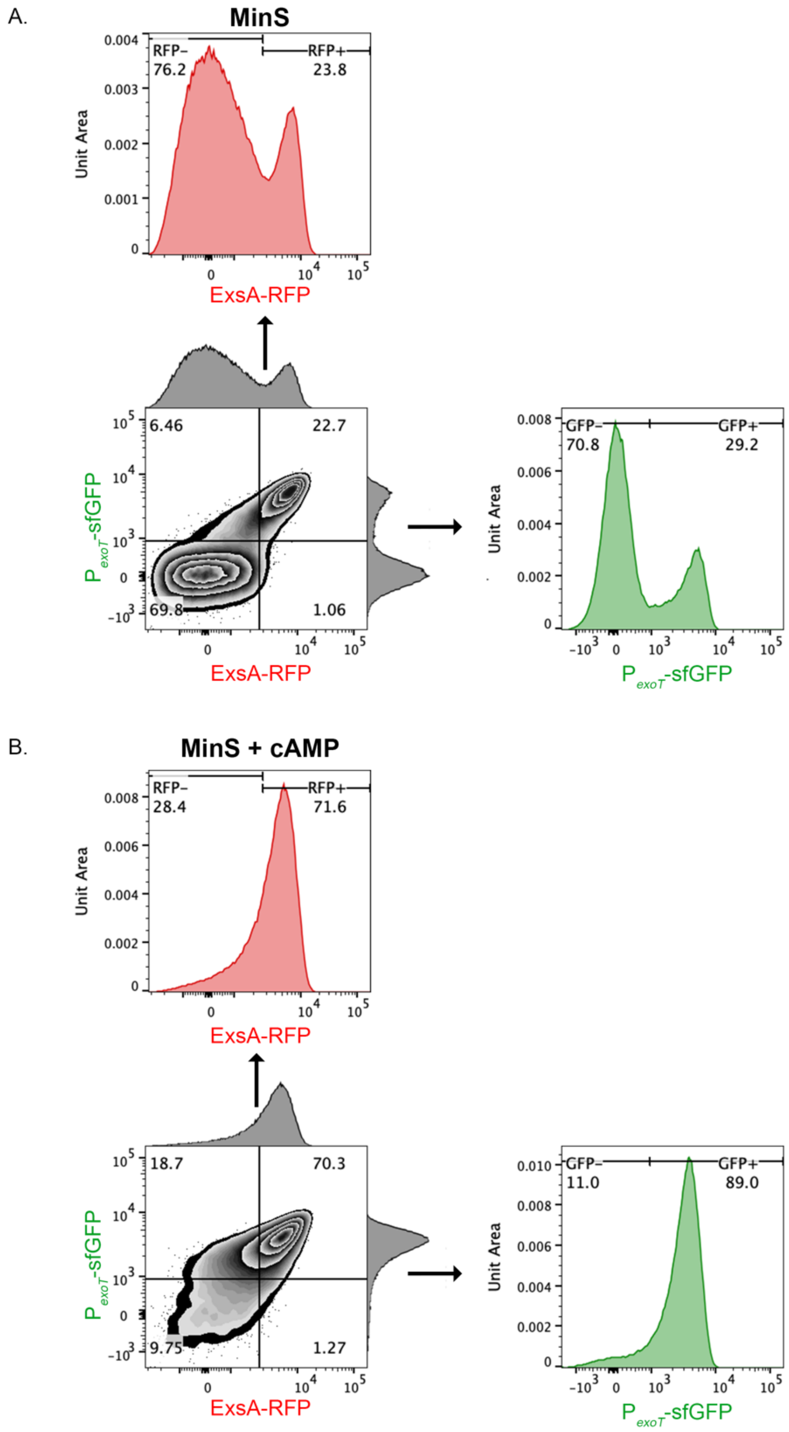

Figure S3. Alternate projections of T3SS-priming in PA14 Dual Reporter. (A) Reproduction of PA14 Dual Reporter 7-hour timepoint in MinS from Figure 1C highlighting the adjunct histograms. ExsA production (red, x-axis) is shown on top of the zebra plot and ExoT transcription (green, y-axis) is shown to the right of the zebra plot. (B) Reproduction of PA14 Dual Reporter 7-hour timepoint in MinS + cAMP from Figure 3C as in (A). Fluorescent gates were established based on a PA14 no fluor control.

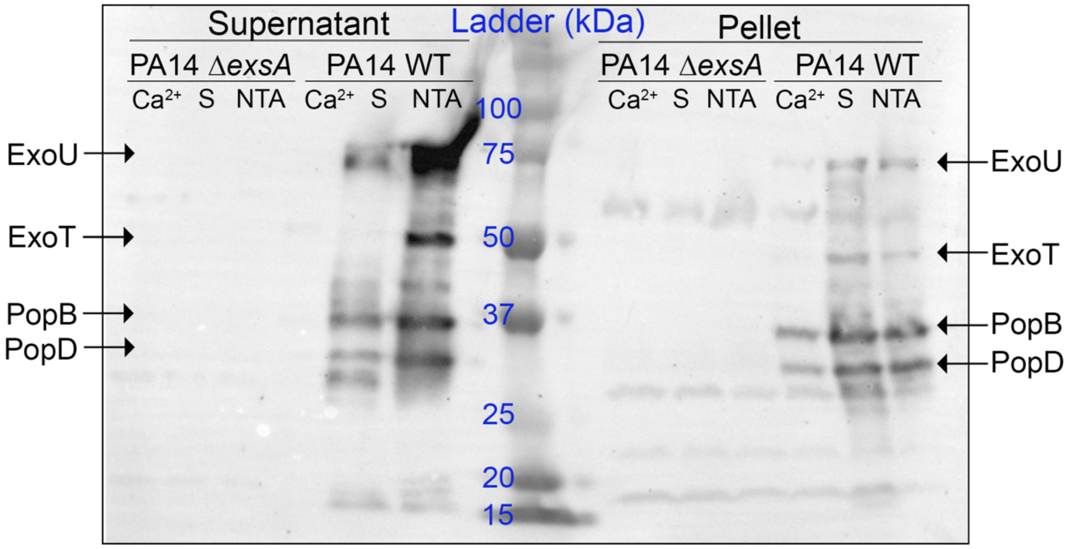

Figure S4: Cell-associated T3SS proteins are produced in MinS + Ca^{2+} but secreted only in the absence of calcium. Cell-associated (pellet) and secreted (supernatant) effector proteins produced by PA14 wild-type or Δ*exsA* bacteria grown in the indicated media were detected by Western blotting with a polyclonal α-T3SS antiserum. A Precision Plus protein ladder was included on the gel; protein molecular weights are shown in kD. "Ca^{2+} ": MinS + Ca^{2+}; "S": MinS; "NTA": MinS + NTA.

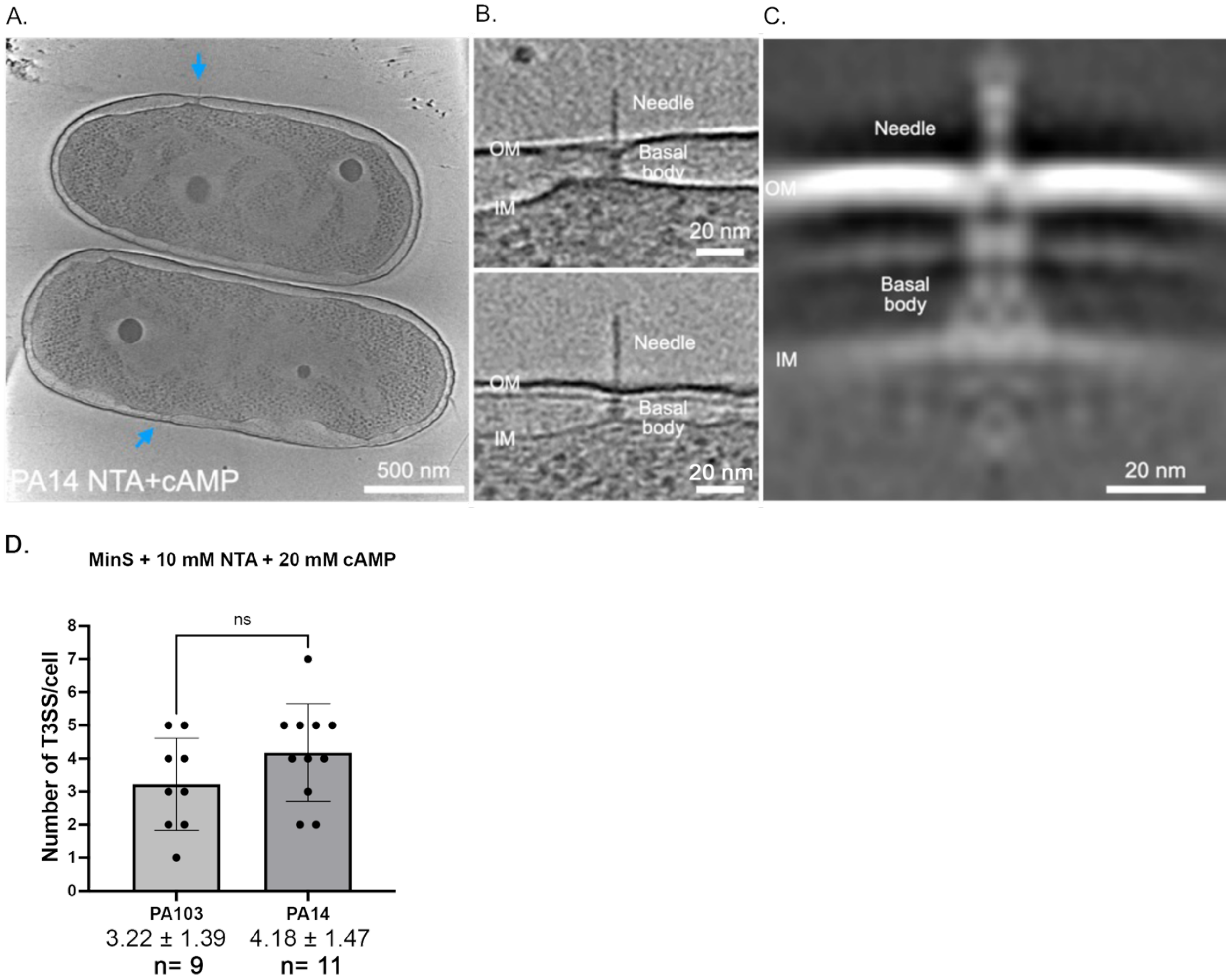

Figure S5: *In situ* structure of the *P. aeruginosa* injectisome determined by subtomogram averaging. (A) A single section of a tomogram showing PA14 cells grown in MinS + 10 mM NTA imaged at a magnification of 6.184 Å physical pixel size. Injectisomes are indicated by blue arrows. (B) Zoomed-in images of injectisomes in tomograms show the needle and basal body in the periplasm. (C) *In situ* structure of the *P. aeruginosa* injectisome, showing the basal body with needle structure. (D) Quantification of T3SS needles per cell in both PA103 and PA14 WT cells grown in MinS + 10 mM NTA + 20 mM cAMP for 7 hours and imaged by cryo-ET. Significance determined by Welch's unequal variances *t*-test.

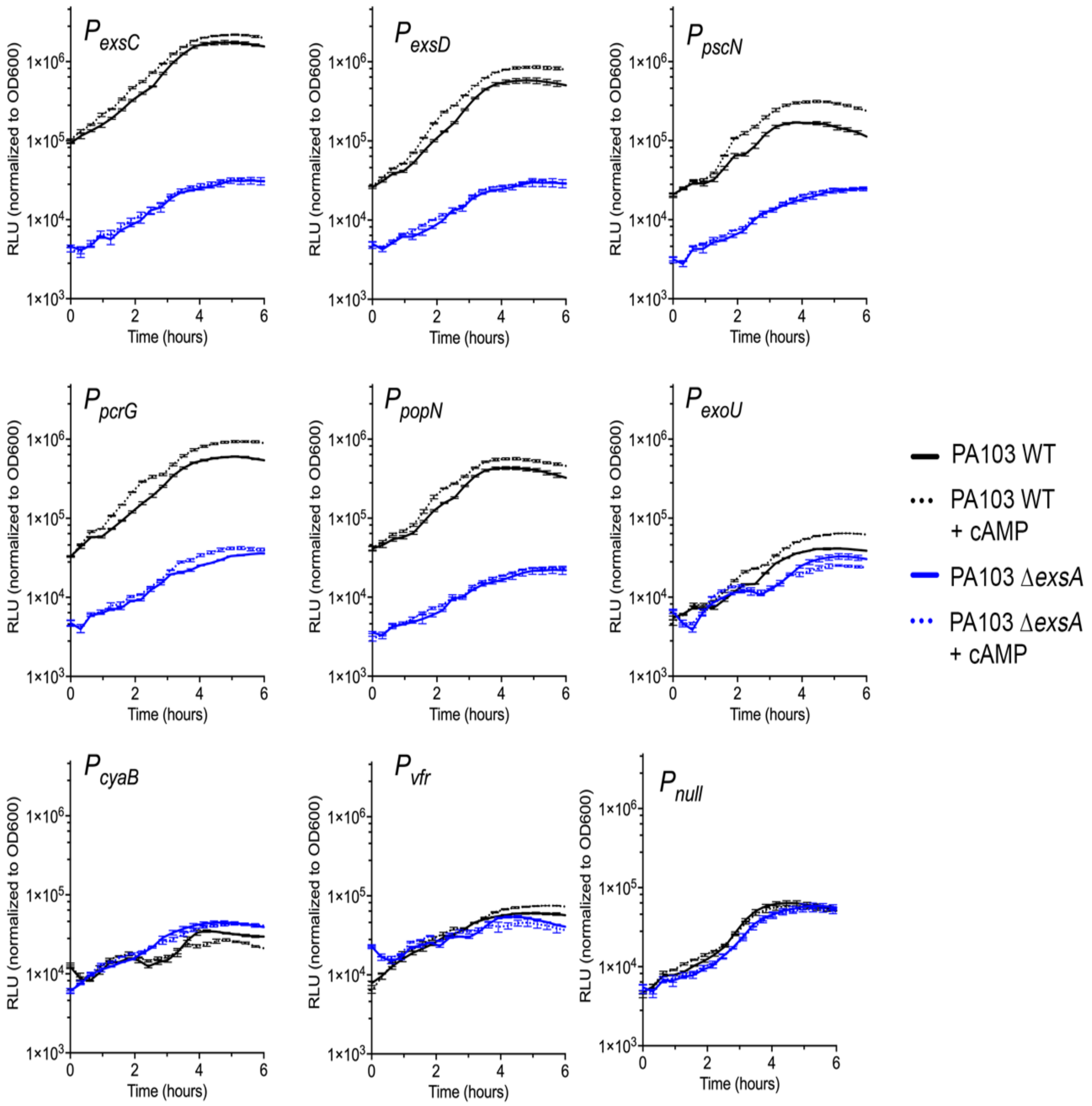

Figure S6. Transcription of T3SS regulatory and structural operons under non-activating conditions requires ExsA and increases with cAMP exposure. PA103 wild-type (black) and PA103 Δ*exsA* (blue) strains, each carrying an *attB* integrated transcriptional *lux* reporter under the control of the indicated promoters were grown planktonically in MinS + Ca^{2+} (solid lines) or MinS + Ca^{2+} + cAMP (dotted lines) in a 96 well plate in a Tecan plate reader for 6hrs with shaking. OD_{600} and luminescence were measured every 10 minutes. Luminescence was normalized to OD_{600} and is shown on a log10 scale. P_{null} is a negative control in which a promoterless *lux* reporter is integrated at *attB*.

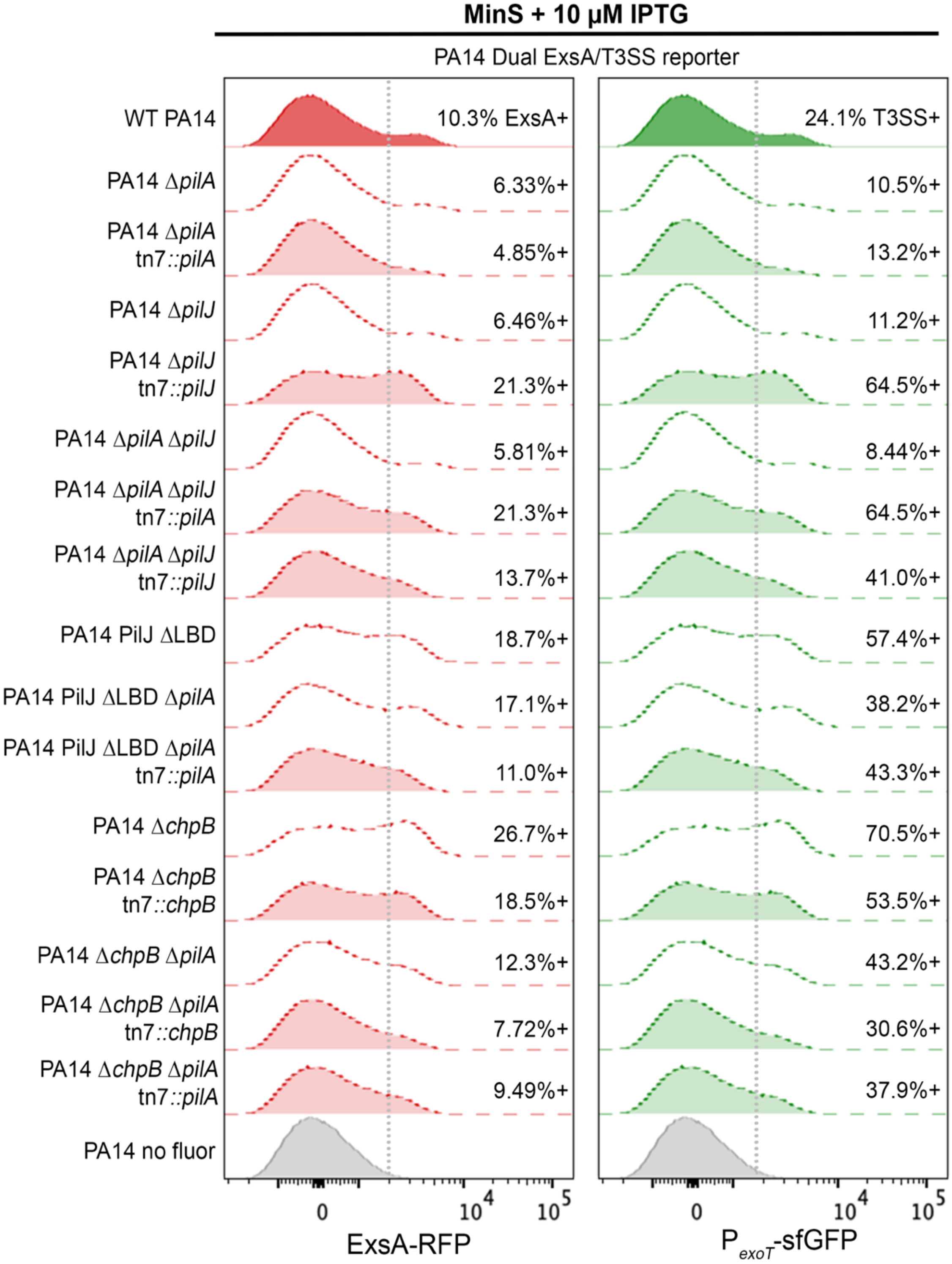

Figure S7. Complementation of T4P mutants. Dual-reporter PA14, PA14 T4P mutants, and PA14 T4P mutants complemented by introducing the indicated gene at the *att*Tn7 site under an IPTG-inducible P_{tac} promoter were grown planktonically in MinS + 10 µM IPTG for 7 hours. Expression of the ExsA-IRES-mTagRFP-t and P_{exoT}-sfGFP T3SS reporters was analyzed by flow cytometry. RFP (left, red) and GFP fluorescence (right, green) are shown separately. Fluorescent gates were established using no fluor *P. aeruginosa* PA14 control (grey line/histogram). Representative histograms shown.

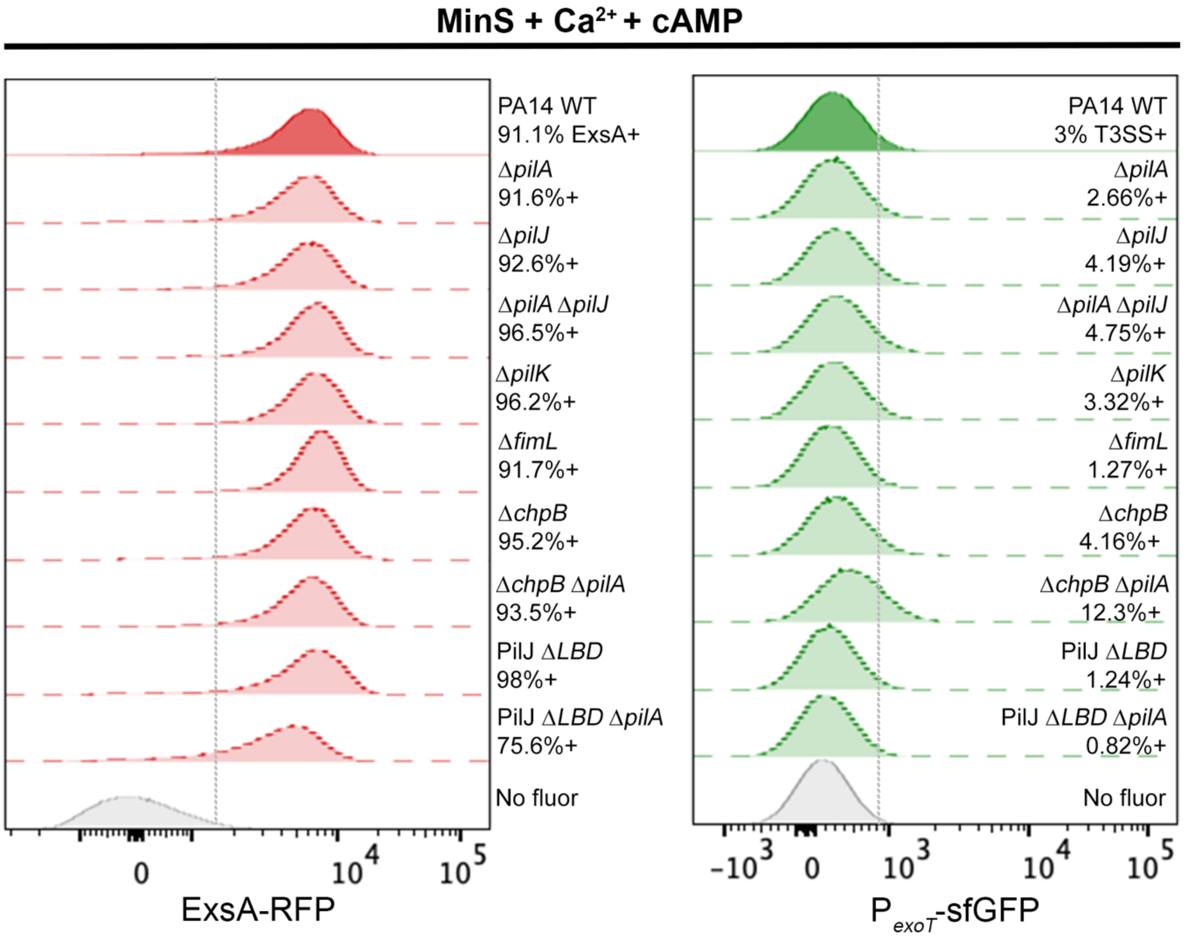

Figure S8. Exogenous cAMP restores priming in Pil/Chp mutants. WT PA14 and the indicated T4P mutants carrying dual ExsA-IRES-mTagRFP-t and P_{exoT}-sfGFP T3SS reporters were grown planktonically in MinS + Ca^{2+} + 20 mM cAMP for 7 hours, then analyzed for RFP (left, red) and GFP fluorescence (right, green) by flow cytometry. Fluorescent gates were established using a no fluor *P. aeruginosa* PA14 control (light grey). Representative histograms shown.

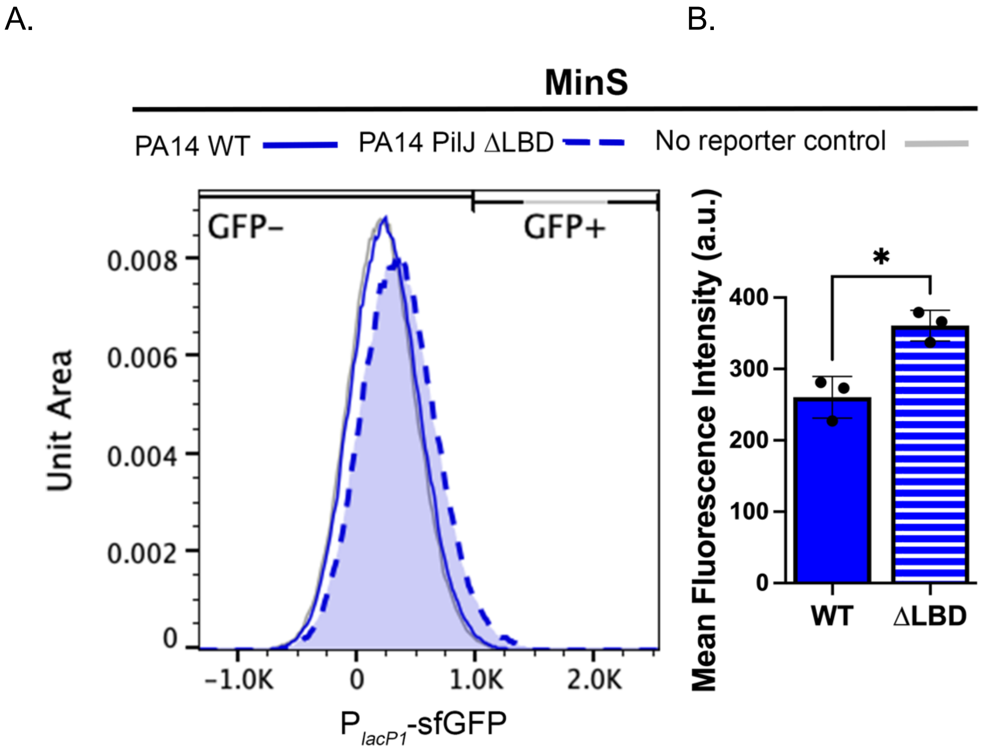

Figure S9. Intracellular cAMP levels are increased in the PilJΔLBD mutant. (A) PA14 (solid blue line) and PA14 PilJΔLBD1-2 (dashed blue line) carrying the *attB*:: P_{lacP1}-sfGFP transcriptional reporter were grown in MinS for 7 hours, then analyzed for GFP fluorescence by flow cytometry. Fluorescent gates were established using a no fluor *P. aeruginosa* PA14 control (grey line). Representative histograms are shown. (B) sfGFP+ mean fluorescence intensity of cells; bars show mean of 3 biological replicates ± S.D. Significance determined by Welch's unequal variances t-test (n = 3). * = P-value < 0.05.

References

1. Simon R, Priefer U, Pühler A. 1983. A Broad Host Range Mobilization System for In Vivo Genetic Engineering: Transposon Mutagenesis in Gram Negative Bacteria. Bio/Technology 1:784-791.
2. Lin CK, Lee DSW, McKeithen-Mead S, Emonet T, Kazmierczak B. 2021. A Primed Subpopulation of Bacteria Enables Rapid Expression of the Type 3 Secretion System in *Pseudomonas aeruginosa*. mBio 12:e0083121-e0083121.
3. Liu PV. 1966. The roles of various fractions of Pseudomonas aeruginosa in its pathogenesis. 3. Identity of the lethal toxins produced in vitro and in vivo. J Infect Dis 116:481-9.
4. Schulert GS, Feltman H, Rabin SDP, Martin CG, Battle SE, Rello J, Hauser AR. 2003. Secretion of the Toxin ExoU Is a Marker for Highly Virulent Pseudomonas aeruginosa Isolates Obtained from Patients with Hospital-Acquired Pneumonia. The Journal of Infectious Diseases 188:1695-1706.
5. Laskowski MA, Osborn E, Kazmierczak BI. 2004. A novel sensor kinase-response regulator hybrid regulates type III secretion and is required for virulence in Pseudomonas aeruginosa. Mol Microbiol 54:1090-103.
6. Cain AK, Nolan LM, Sullivan GJ, Whitchurch CB, Filloux A, Parkhill J. 2019. Complete Genome Sequence of *Pseudomonas aeruginosa* Reference Strain PAK. Microbiol Resour Announc 8.
7. Wolfgang MC, Lee VT, Gilmore ME, Lory S. 2003. Coordinate regulation of bacterial virulence genes by a novel adenylate cyclase-dependent signaling pathway. Dev Cell 4:253-63.
8. Fulcher NB, Holliday PM, Klem E, Cann MJ, Wolfgang MC. 2010. The *Pseudomonas aeruginosa* Chp chemosensory system regulates intracellular cAMP levels by modulating adenylate cyclase activity. Molecular Microbiology 76:889-904.
9. Hoang TT, Karkhoff-Schweizer RR, Kutchma AJ, Schweizer HP. 1998. A broad-host-range Flp-FRT recombination system for site-specific excision of chromosomally-located DNA sequences: application for isolation of unmarked *Pseudomonas aeruginosa* mutants. Gene 212:77-86.
10. Fürste JP, Pansegrau W, Frank R, Blöcker H, Scholz P, Bagdasarian M, Lanka E. 1986. Molecular cloning of the plasmid RP4 primase region in a multi-host-range tacP expression vector. Gene 48:119-131.
11. Pankratz D, Gomez NO, Nielsen A, Mustafayeva A, Gür M, Arce-Rodriguez F, Nikel PI, Häussler S, Arce-Rodriguez A. 2023. An expanded CRISPR–Cas9-assisted recombineering toolkit for engineering genetically intractable *Pseudomonas aeruginosa* isolates. Nature Protocols 18:3253-3288.
12. Shanks RM, Caiazza NC, Hinsa SM, Toutain CM, O'Toole GA. 2006. Saccharomyces cerevisiae-based molecular tool kit for manipulation of genes from gram-negative bacteria. Appl Environ Microbiol 72:5027-36.
13. Choi K-H, Schweizer HP. 2006. mini-Tn7 insertion in bacteria with single *att*Tn7 sites: example *Pseudomonas aeruginosa*. Nature Protocols 1:153-161.
14. Hall A, Donohue T, Peters J. 2023. Complete sequences of conjugal helper plasmids pRK2013 and pEVS104. MicroPubl Biol 2023.
